# The mechanochemical activation of a pyrimidine dimer

**DOI:** 10.1039/d6sc02165d

**Published:** 2026-04-17

**Authors:** Xiang Gao, Gurudas Chakraborty, Felix Joel Urhahne, Marcus Lantzius-Beninga, Regina Lennarz, Jilin Fan, Kara Stappert, Jan Meisner, Robert Göstl, Andreas Herrmann

**Affiliations:** a Institute of Technical and Macromolecular Chemistry, RWTH Aachen University Worringerweg 2 52074 Aachen Germany herrmann@dwi.rwth-aachen.de; b DWI – Leibniz-Institute for Interactive Materials Forckenbeckstraße 50 52056 Aachen Germany chakraborty@dwi.rwth-aachen.de; c Institute for Physical Chemistry, Heinrich Heine University Düsseldorf Universitätsstraße 1 40225 Düsseldorf Germany; d Department of Chemistry and Biology, University of Wuppertal Gaußstraße 20 42119 Wuppertal Germany

## Abstract

The cyclobutane motif is a versatile force-reactive moiety enabling diverse mechanophores and their associated functions in polymer mechanochemistry. For example, the cyclobutane core has been applied in the context of stress-responsive polymers, self-healing materials, and self-sensing nanocomposites. However, leveraging the cyclobutane structure for the development of nucleobase- and nucleoside-derived mechanophores remains unexplored. Here, we introduce a pyrimidine dimer mechanophore based on a cyclobutane core (CPD), formed *via* photoinduced [2 + 2] cycloaddition of thymine under ultraviolet B (UVB) irradiation. Upon ultrasound exposure, the polymer-centered CPD undergoes formal cycloelimination. To better understand the individual mechanochemical contributions of the four possible CPD stereoisomers upon force input, we employ the CoGEF and FM-PES methods and compare the results. Experimental and computational methods suggest that the *syn*-diastereomers cleave preferentially compared to their *anti*-counterparts under mechanical force.

## Abstract 2

The mechanochemical activation of a cyclobutane-based mechanophore formed by pyrimidine photodimerization, revealing different reactivities of the individual isomers.

## Graphical abstract

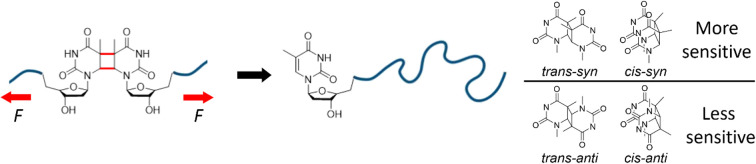

## 1 Introduction

Polymer mechanochemistry has rapidly advanced as a field focused on harnessing mechanical energy to drive specific chemical transformations.^1,2^ In this context, ultrasound serves as a key source of mechanical force, widely used in solution-phase polymer systems.^3^ Upon the collapse of cavitation bubbles, ultrasound generates elongational flow that overstretches segments of reasonably long polymer chains, eventually leading to bond scission.^4,5^ Building on this foundation, a wide variety of mechanochemically responsive motifs (mechanophores) have been developed^6^ and embedded in synthetic polymers to trigger distinct functions in response to mechanical force, including catalysis,^7–9^ initiating cross-linking,^10,11^ releasing small molecules,^12–20^ or visually reporting material damage.^21–25^

These functions have also been uniquely realized using biomacromolecules, with demonstrations of ultrasound-induced drug activation by covalent and noncovalent bond cleavage within nucleic acid scaffolds, introducing a new research domain of sonopharmacology.^26^ This polyaptamer-based deactivation and ultrasound-triggered activation approach was also applied to activate thrombin, enabling it to catalyze the conversion of fibrinogen into fibrin.^27^ Additional advances include sonocontrolled optical and catalytic activity of genetically engineered proteins,^28^ mechanochemical activation of DNAzymes,^29^ and fracture detection in bio-glues using fluorescent-protein-based optical force probes.^30^

Within the specific context of sonopharmacology, ultrasound-responsive mechano-nanoswitches have also been developed,^31^ consisting of gold nanoparticles (AuNPs) functionalized with a defined number of DNA strands on their surfaces. These nanoparticles form dimers by Watson–Crick base pairing, enabling the incorporation of anticancer drugs into the double helix. Upon ultrasound application, the drugs are liberated, leading to killing of cancer cells *in vitro*. More importantly, the applicability of polymer mechanochemistry principles has recently been demonstrated *in vivo*.^32^ A universal polynucleotide framework was developed, enabling the binding and release of therapeutic oligonucleotides, both DNA and RNA, using biocompatible medical-imaging ultrasound as the mechanical trigger. However, hydrogen-bonding-based caging strategies are often susceptible to thermal denaturation and enzymatic degradation. To address these limitations, an alternative approach involves caging oligonucleotides by exploiting covalent linkages between adjacent nitrogenous bases along the same strand, such as through the formation of CPDs, thereby exploring the mechanochemical reactivity potential of nucleobase- and nucleoside-derived motifs.

CPDs are the primary DNA lesions formed upon exposure to UVB light. They result from a [2 + 2] cycloaddition between the C5–C6 double bonds of adjacent pyrimidine bases.^33–35^ Although such damage can be reversed through photorepair mechanisms, such as photolyase-mediated electron transfer that cleaves the four-membered ring,^36,37^ the application of mechanical force to induce CPD ring-opening remains unexplored. Investigating the mechanochemical response of CPDs under ultrasound could not only establish their function as mechanophores but also open new avenues for their use in biologically relevant systems.

**Scheme 1:**
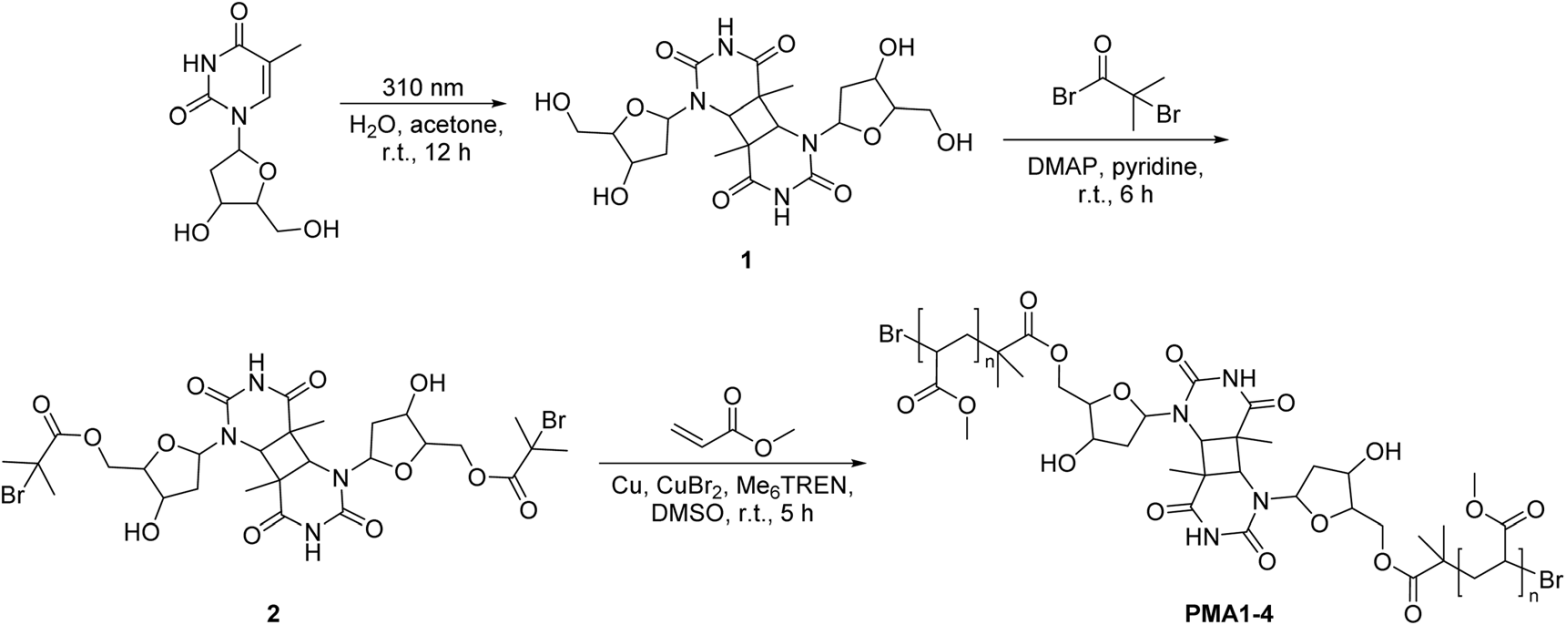
The cycloaddition reaction under UVB light and Cu(0)-mediated CRP.

Indeed, cyclobutane-core mechanophores are well-established in polymer mechanochemistry^38–40^ underpinning the plausibility of this hypothesis. Therefore, we employ synthetic polymers rather than long-chain DNA as carriers for mechanical force transmission. Our experimental design centers on synthesizing CPDs *via* UVB-induced [2 + 2] cycloaddition of thymine^41^ and incorporating them into linear poly(methyl acrylate) (PMA) chains of varying molar masses (*M*_n_ ∼33–104 kDa) using Cu(0)-mediated controlled radical polymerization (CRP, Scheme 1).^42,43^ The mechanochemical behavior of these polymers was then probed under ultrasound exposure. We applied established mechanochemical conditions in this work (20 kHz ultrasound and linear PMA polymers), paving the way for future work to exploit medically relevant ultrasound, such as focused ultrasound with a mechanical index below 1.9 – the FDA approval limit.^44,45^ Gel permeation chromatography (GPC) revealed distinct peaks corresponding to half the original molar mass, confirming ultrasound-induced chain scission. By varying the ultrasound intensity (3–14 W cm^−2^), we quantified the corresponding rate constants for ultrasound-induced polymer cleavage (Table 1). To determine the site of cleavage, nuclear magnetic resonance (NMR) spectroscopy was employed to detect characteristic olefinic proton resonances before and after ultrasonication, providing evidence of bond rupture at the CPD site. To complement these findings, constrained geometries simulate external force (CoGEF) and force-modified potential energy surface (FM-PES) computations were performed on the four CPD isomers (*trans*–*syn*, *cis*–*syn*, *trans*–*anti*, and *cis*–*anti*) to evaluate their ring-opening force profiles and associated energy requirements.^46,47^ Collectively, this study not only establishes CPDs as a new class of mechanophores through comprehensive experimental and computational analyses but also introduces a versatile model for investigating nucleobase- and nucleoside-derived mechanochemistry, possibly also applicable to other photodimers, including cytosine dimers.^48^

## 2 Results and discussion

### 2.1 UV-induced [2 + 2] cycloaddition for CPD formation

**Fig. 1:**
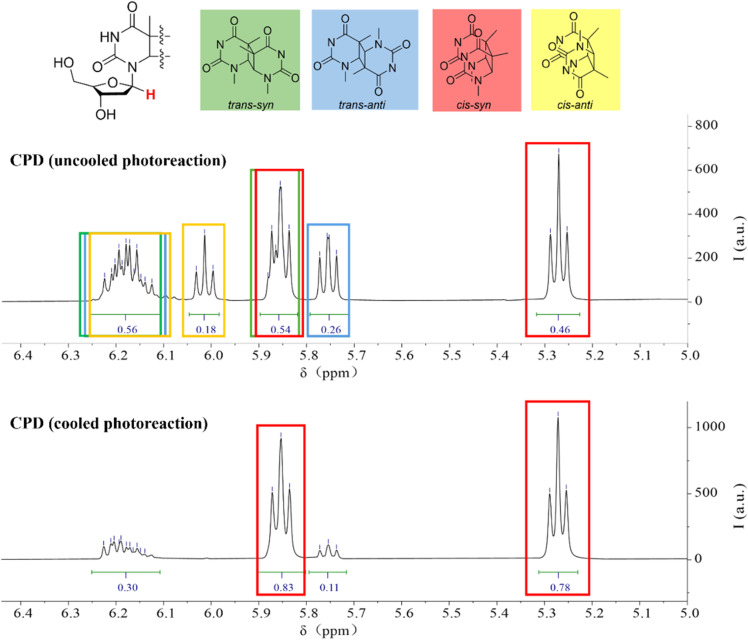
The ^1^H NMR spectrum of the 1-position H of thymidine in CPD. The cooled photoreaction was performed at 10 °C and the uncooled reaction was performed at room temperature.

Thymine generates a CPD structure under UVB light irradiation. It is noteworthy that this reaction strongly depends on the wavelength of UV light. In fact, reports indicate that under UVA or UVC irradiation, thymine produces 6–4 photoproducts.^49,50^ Moreover, the CPD structures formed under UV light are predominantly of the *cis*–*syn* configuration.^51^ Based on this, we irradiated thymidine with a 310 nm UVB source to obtain CPD products (Scheme 1). In the ^1^H NMR spectra, we observed the presence of four different structural isomers (Fig. 1).

The chemical shift of the 1-position protons in the dimer experiences mutual coupling, and in different isomers, the chemical shifts also vary. Based on this, we were able to determine the content of the different structural isomers. The characteristic peaks for *trans*–*syn* appeared at *ca.* 6.20 ppm and 5.85 ppm, for *trans*–*anti* at *ca.* 6.20 ppm and 5.75 ppm, for *cis*–*syn* at *ca.* 5.85 ppm and 5.25 ppm, and for *cis*–*anti* at *ca.* 6.20 ppm and 6.00 ppm. Among the four isomers, we found that the *cis*–*syn* structure was the major product, albeit with a relatively low yield of only 46% (Fig. 1, top). In order to better mimic the natural formation of CPD, we attempted to cool the reaction mixture by immersing it in cold water, aiming to mitigate the potential impact of prolonged exposure to high temperatures caused by continuous light irradiation. As a result, we observed a significant increase in the content of the *cis*–*syn* isomer, reaching up to 78% (Fig. 1, bottom). While we still observed the formation of the other three isomers in low yield, our primary emphasis, however, was not to isolate all four species, but to directly investigate the effect of mechanical force on the natural photoproducts of the UVB-induced dimerization reaction, with a particular focus on monitoring CPD cleavage under ultrasound.

### 2.2 Synthesis and ultrasonication of CPD-containing polymers

After obtaining the CPD structure, we further synthesized initiator 2 and employed Cu(0)-mediated CRP to synthesize various linear polymers with different molar masses (Scheme 1). These included poly(methyl acrylate) PMA1 (*M*_n_ = 104 kDa, *Đ*_M_ = 1.18), PMA2 (*M*_n_ = 71 kDa, *Đ*_M_ = 1.27), PMA3 (*M*_n_ = 49 kDa, *Đ*_M_ = 1.24), and PMA4 (*M*_n_ = 33 kDa, *Đ*_M_ = 1.31) (Fig. S10–S13). The polymerization reaction was initiated on both sides of the CPD mechanophore under identical conditions, ensuring that the mechanophore structure was positioned in the center of the polymer. This approach accounts for the maximum mechanochemical reactivity in the subsequent ultrasonic experiments.^4^

**Fig. 2:**
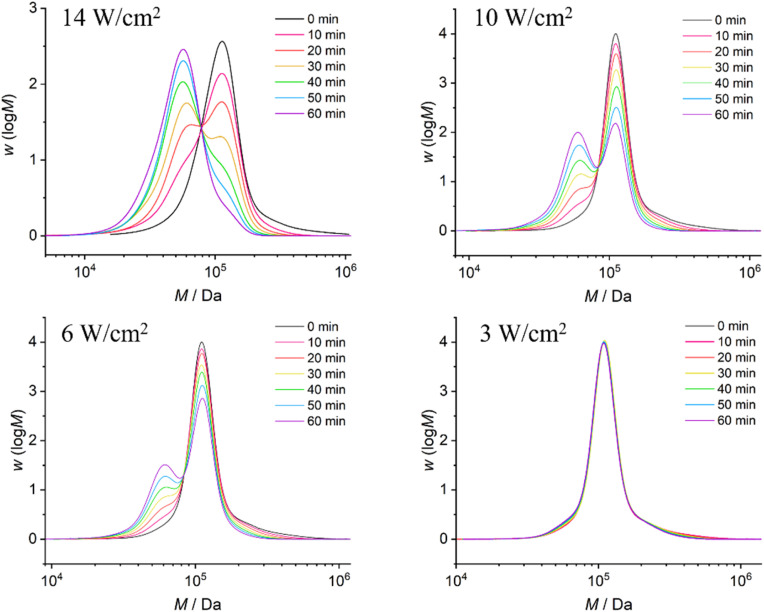
GPC traces of PMA1 with an initial *M*_n_ of 104 kDa after ultrasonic treatment at different intensities.

**Fig. 3:**
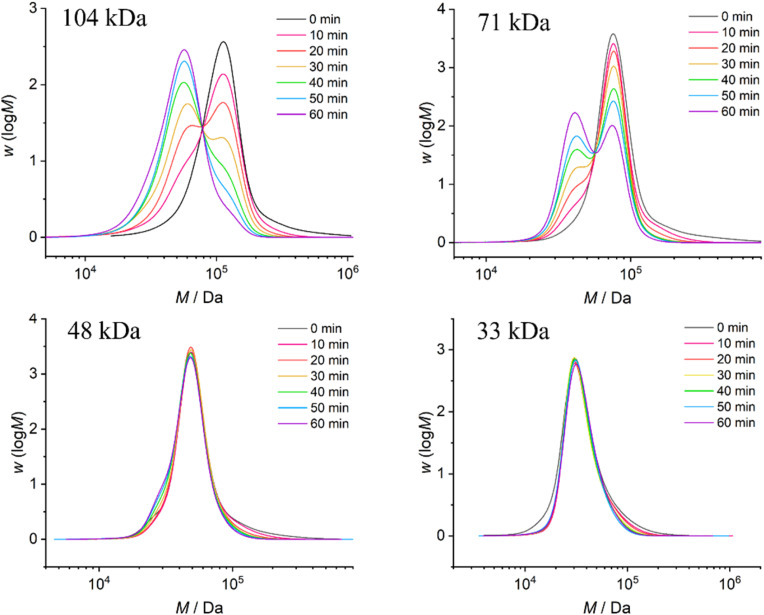
The GPC traces of PMA1–4 with varying initial *M*_n_ after ultrasonic treatment at an intensity of 14 W cm^−2^.

The ultrasonication experiments were conducted using a 20 kHz immersion probe sonicator, with the solution adequately cooled using ice water. Samples were taken every 10 min for GPC measurements to monitor the molar mass distribution after different durations of ultrasonication (Fig. 2 and 3). Aligning with the positioning of the mechanophore at the center of the polymer chain, we observed the formation of a new peak corresponding to half of the initial molar mass. Furthermore, we clearly observed that with decreasing ultrasound intensities, ranging from 14 W cm^−2^ to 3 W cm^−2^, there was a significant and expected reduction in the rate of polymer chain rupture (Fig. 2). Analogously, we observed the same trend for polymers with decreasing molar masses at constant intensity (14 W cm^−2^) (Fig. 3).

To quantify the influence of molar mass and ultrasound intensity on the mechanophore cleavage rates, we calculated the apparent scission rate constant for this mechanophore (the mixture of all isomers). We used a calculation method (eqn (1)) based on the approach of Malhotra, in which *k*′= *k*/*M*_0_, where *M*_0_ is the molar mass of the monomer unit, *M*_i_ is the initial number average molar mass of the polymer, *M*_*t*_ is the number average molar mass of the sonicated sample at time *t*, and *k* is the rate constant of polymer cleavage with initial molar mass *M*_i_.^52,53^ Additionally, Moore and coworkers developed and efficiently applied this formula to calculate the rate constants for cyclobutane-based mechanophores.^38^1
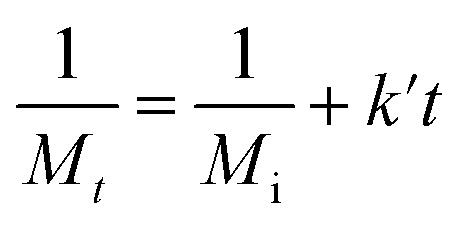


**Fig. 4:**
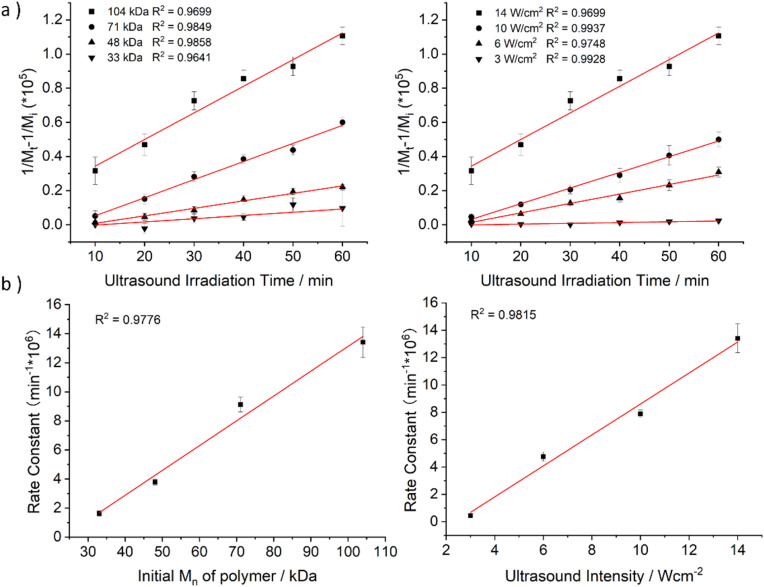
Experimentally determined rate constants of polymer cleavage as a function of initial polymer molar mass. (a) The fitting line of 1/*M*_*t*_ − 1/*M*_i_ against ultrasonication time, where the slope of the line represents the ultrasonic rate constant *k′*. (b) The fitting line of the rate constant *k′* against the initial number average molar mass of the polymer *M*_i_ or ultrasonic intensity.

The *M*_n_ data obtained from GPC measurements for products under different ultrasonic conditions can be fitted to the above-mentioned formula (Fig. 4a). The calculated rate constants *k*′ under different intensities and molar masses are presented in Tables 1 and 2. Furthermore, the cleavage rate constant *k*′ also demonstrates a linear relationship (Fig. 4b), providing strong evidence that ultrasound waves activate the cleavage process of CPD mechanophores.

**Table 1:** Rate constants of PMA1 (104 kDa) at different ultrasound intensities

Ultrasound intensity *I*_P_/W cm^−2^	Rate constant *k*/10^−6^ min^−1^
14	13.4
10	7.90
6	4.76
3	0.40

**Table 2:** Rate constants of different *M*_i_ at 14 W cm^−2^ US intensity

Initial molar mass *M*_i_/kDa	Rate constant *k*/10^−6^ min^−1^
104	13.4
71	9.12
49	3.78
33	1.63

However, the ultrasound-activated mechanophore cleavage competed with the random chain scission of the polymer backbone. While we have already demonstrated the cleavage of CPD mechanophores under ultrasound, it is crucial to further prove the exact cleavage positions. We employed NMR as a non-optical characterization method to detect the formation of double bond H signals, considering the non-fluorescent character of the designed mechanophore. Although observing a specific proton signal throughout the entire polymer can be challenging due to significant interference, there are no other signals in the vicinity of the double bond proton signals in the PMA, allowing us to detect the formation of double bonds after ultrasound exposure.

**Fig. 5:**
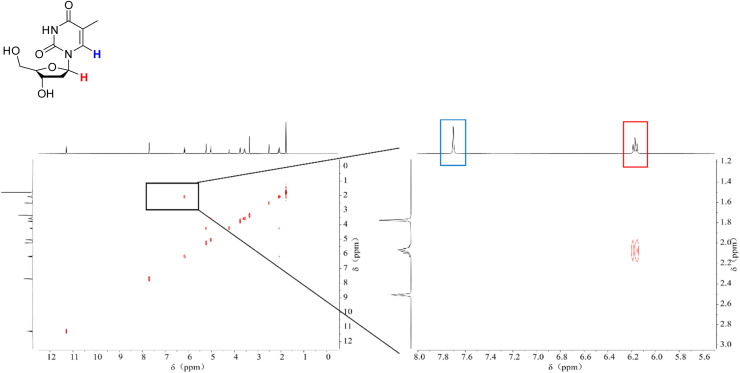
The HH-COSY spectrum of thymine molecules, with a magnified section showing the correlation of the double bond H (blue) and the 1-position H (red) individually.

**Fig. 6:**
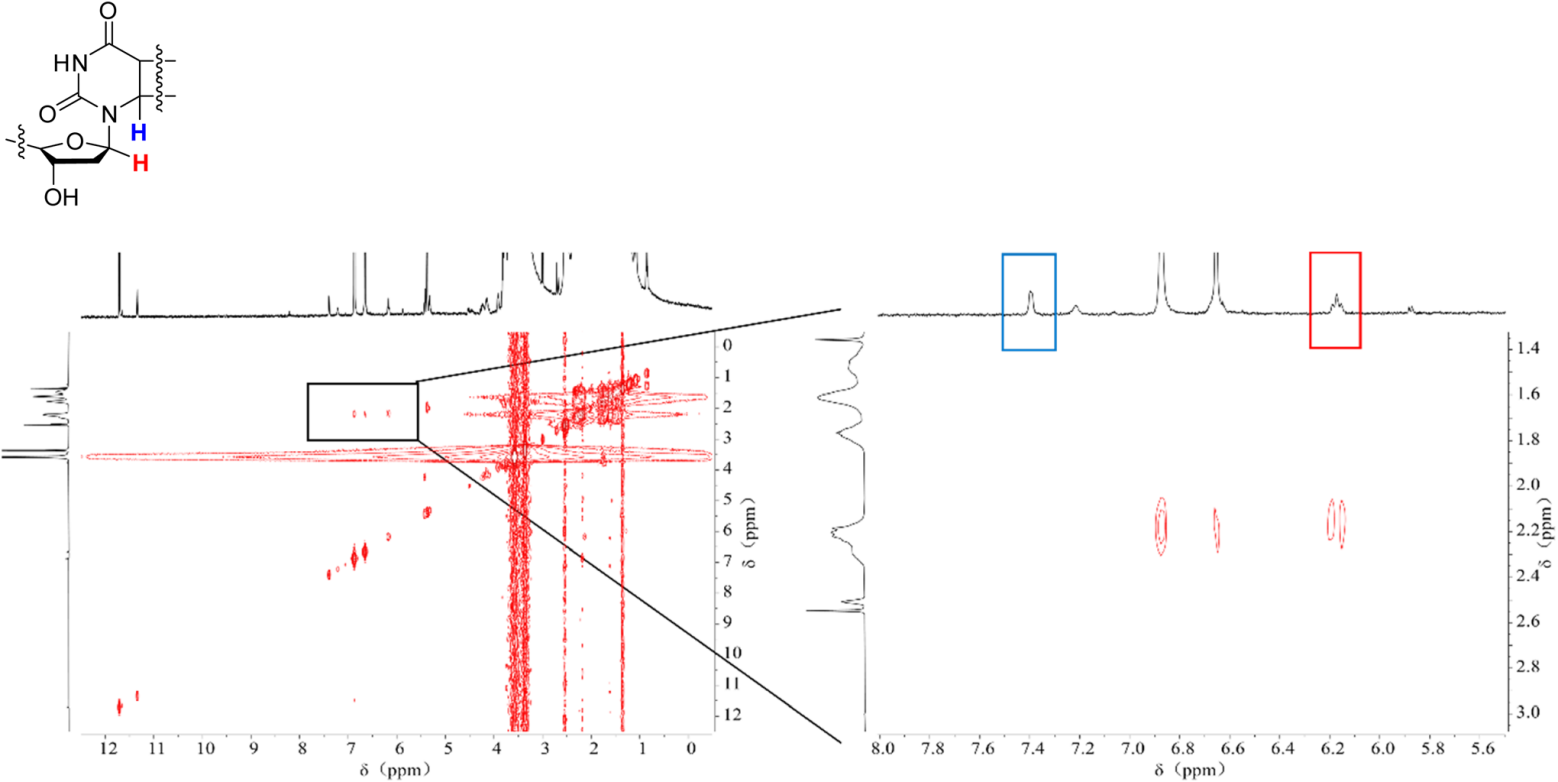
The HH-COSY spectrum of PMA1 after ultrasound exposure, with a magnified section showing the correlation of the double bond H (blue) and the 1-position H (red) individually. The intermediate appearance of the double peak corresponds to the residual BHT in the polymer.

**Fig. 7:**
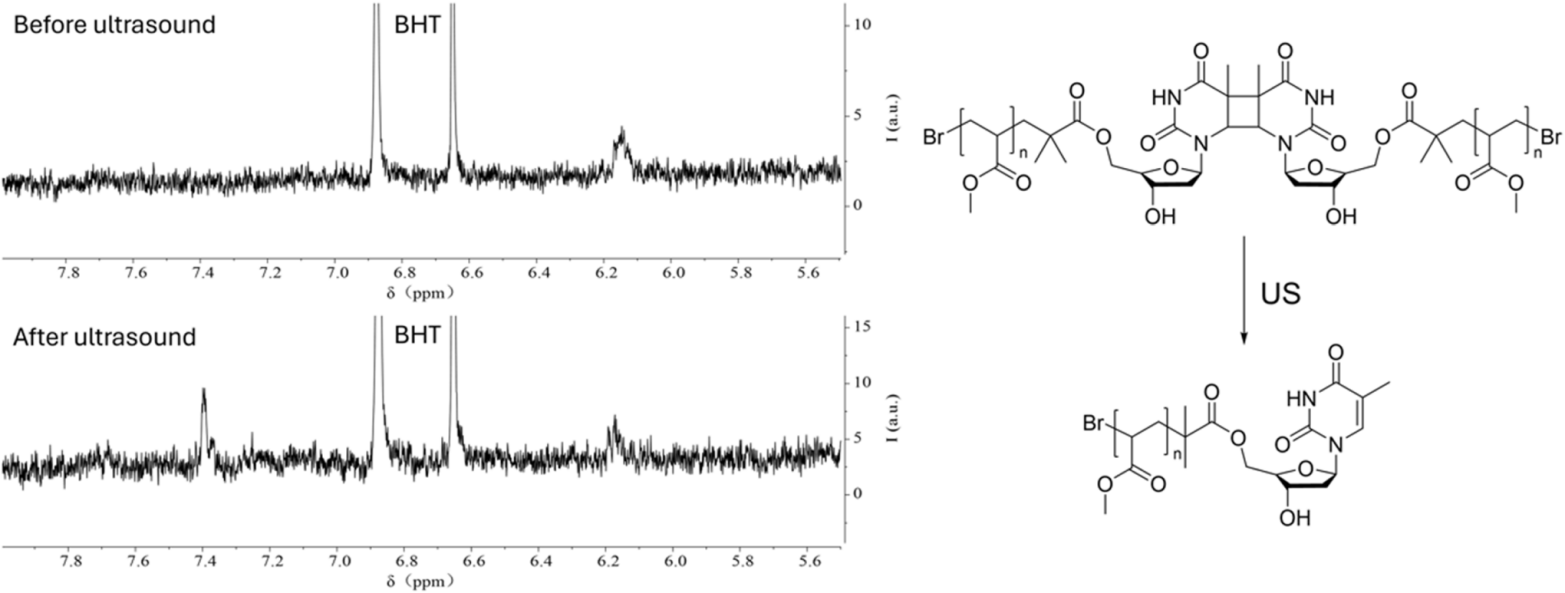
The ^1^H NMR spectrum of PMA1 before and after ultrasonication, showing the reappearance of the double bond H signal after 30 min of ultrasonication. BHT was used as the internal standard.

We conducted NMR measurements on the individual thymidine molecule and PMA1 polymers in DMSO-d_6_ solution before and after ultrasound irradiation. In the HH-COSY spectrum of the thymidine molecule (Fig. 5), the double bond proton signal (blue) is at 7.7 ppm, and the 1-position proton (red) signal is at 6.2 ppm. We also observed coupling relationships between the 1-position and 2-position proton signals. In the COSY spectrum of PMA1 after ultrasound exposure (Fig. 6), we could similarly determine the signal for the 1-position proton based on the coupling relationships. Due to the differences in chemical environments between the solvent and the polymer, there was a slight shift in the double bond proton signal. When comparing the NMR spectra before and after ultrasound exposure (Fig. 7), we found that there was no double bond proton signal before the CPD mechanophore was cleaved by ultrasound. After ultrasound irradiation, the double bond proton signal reappears. Based on this, we can confirm that the cleavage position of the mechanophore is primarily at the cyclobutane site, and the structure after cleavage restores the double bond structure of thymidine.

### 2.3 Experimental and computational investigations of the isolated isomers

**Scheme 2:**
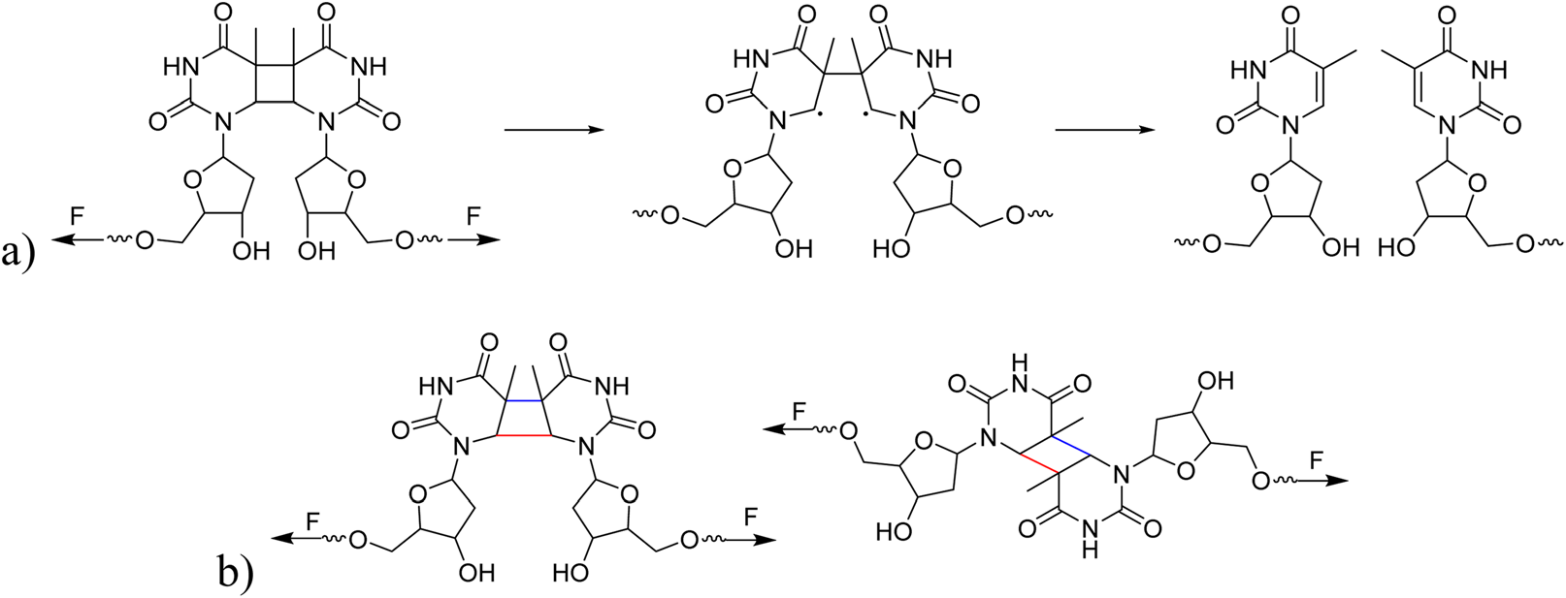
(a) The process of CPD mechanophore cleavage induced by mechanical force. (b) The potential for different cleavage outcomes of *syn*- and *anti*-conformations of CPD mechanophores under mechanical force. The bonds marked in red indicate the bond at which the first C–C scission occurs, while the bonds marked in blue indicate the second C–C scission.

Hereafter, we investigated whether different isomeric structures of CPD exhibit significant differences in their response to ultrasound. The mechanical cleavage of *syn*-configured cyclobutane-type mechanophores generally involves two main processes: single bond cleavage, leading to the formation of a diradical intermediate, followed by rapid diradical-mediated ring opening and formation of a diene (Scheme 2a).^3^ Upon the first C–C bond breaking, the second C–C bond is strongly activated. The responsiveness to force depends on the linker and on the *cis*/*trans* configuration. The mechanical cleavage of *anti*-configured cyclobutane is expected to require higher forces, because the two bonds to be broken are not aligned parallel to the tensile axis and, thus, the force is transduced less directly (Scheme 2b).^3^ We isolated the *cis*–*syn*, *cis*–*anti* (−), *cis*–*anti* (+), and *trans*–*anti* isomers of CPD by preparative high-performance liquid chromatography (HPLC) (the *trans–syn* isomer is only formed in traces) and functionalized the *cis*–*syn* and *trans*–*anti* isomers with initiators for CRP. The resulting PMA polymers with comparable *M*_n_ (*cis*–*syn*: 51.0 kDa, *trans*–*anti*: 50.2 kDa, Fig. S12) were subjected to ultrasonication at low intensity to resolve any differences in reactivity of the isomers (0–30 min, 20% amplitude, 3 W cm^−2^) and the scission rates were determined as demonstrated above (*vide supra*). The analysis of the ultrasound-induced bond scission by GPC showed activation of the *cis*–*syn* PMA, while the scission rate of the *trans*–*anti* PMA was the same as for the control PMA. These findings indicate a reactivity difference of the individual isomers (Fig. S20–S22). We further investigated the mechanochemical behaviour of the isomers by computational methods for a complementary comprehension of the responsiveness of these isomers to the applied force.

We performed CoGEF^46^ and FM-PES^54^ calculations on the four isomeric models of CPD at the B3LYP-D3/6-31G* level^55–61^ of theory. Mechanical force was applied at the 5′ end of deoxyribose, propagating through the entire deoxyribose molecule onto the cyclobutane structure. While CoGEF is a straightforward and highly accessible method to compute the influence of force on molecular systems, it purely relies on optimized geometric structures of increased geometric displacement. Therefore, although dissociation energies and rupture forces *F*_max_ are available, reaction pathways are not available, which means that reactive intermediates might be missed. Furthermore, the values of the rupture forces obtained are too large (compared to the experiment) because the method does not take thermal effects into account.^62,63^ Nevertheless, due to its low computational effort, it is well suited as a method to investigate the influence of mechanical force on a novel mechanophore. The FM-PES computations rely on a potential energy surface, which is distorted (force-modified), so that minimum structures, transition state structures, and reaction paths can be obtained for different forces under investigation. This significantly more expensive approach allows the computation of barrier heights depending on the applied force and, thus, enables mechanochemical and purely thermal reactions to be treated on equal footing.^54,62^

**Fig. 8:**
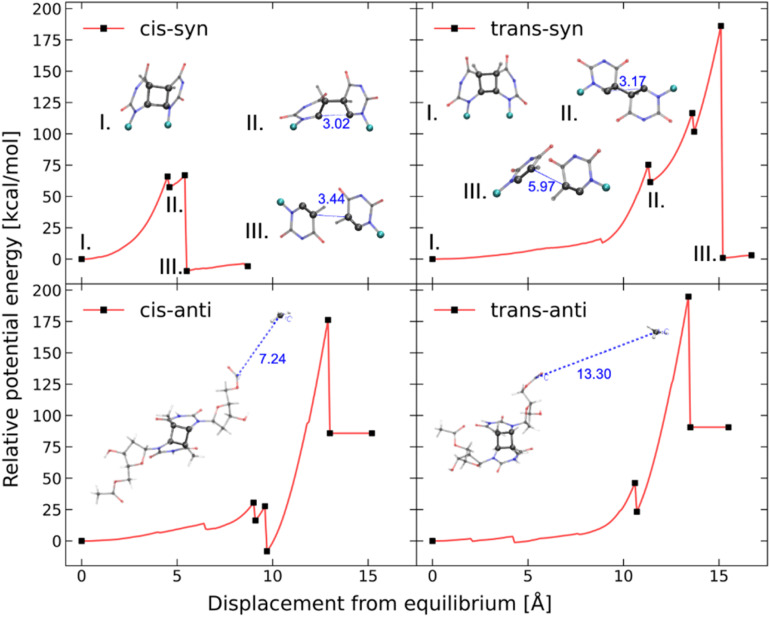
CoGEF results for the four CPD mechanophore isomers investigated. For the syn-CPDs, the structures without displacement (I), just after the first C–C bond breaking (II), and after the second C–C bond breaking (III) are displayed. For the *anti*-CPDs, only the geometry after the dissociation of the CH_3_ group is displayed. The other black dots represent conformational changes induced by the mechanochemical stretching.

Our CoGEF results show significant differences for the four isomeric structures (Fig. 8): both *syn*-CPDs are predicted to open in a two-step fashion with similar values of 
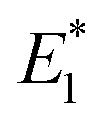
 for the first C–C bond breaking (Fig. 8, upper panel). The *cis*–*syn* intermediate then requires less energy to break the second C–C bond, as the bond is already activated. However, the value of 
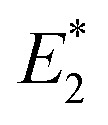
 for the *trans*–*syn* intermediate is dubiously large. The reason for this is that the *trans*–*syn* intermediate twists, thereby reducing the force transduction to the second scissile bond. This large value of 
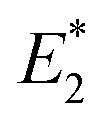
 does not relate to a barrier that must be overcome for the second reaction step, but rather reflects the amount of energy introduced into the whole system during the stretching process. In the case of both *anti*-configured CPDs, one of the terminal CH_3_ groups breaks away at the end of the CoGEF simulations (Fig. 8, lower panel). This could, in principle, be regarded as a lack of mechanochemical activation, but the CoGEF method has been shown to fail to predict *anti*-configured cyclobutane mechanophores in the past.^47^

**Fig. 9:**
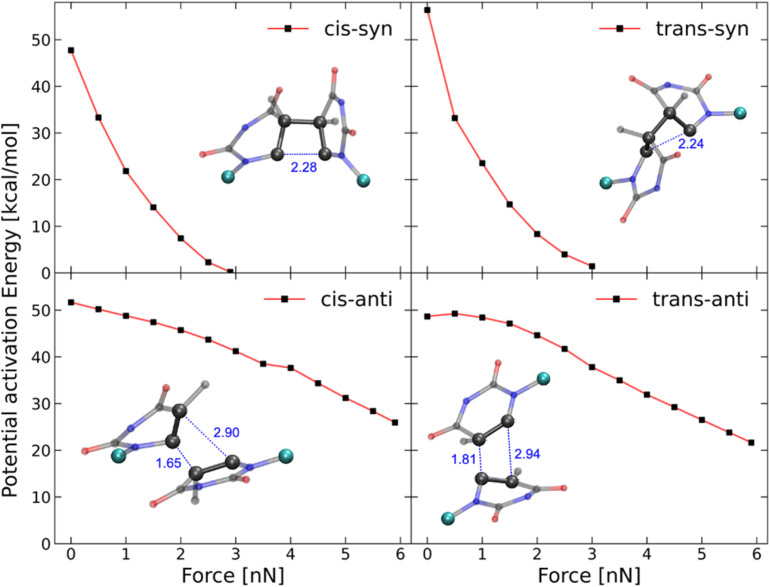
Barrier heights of the first bond breaking of the four CPD mechanophore isomers investigated computed with the FM-PES method. The displayed geometries show the transition state structures with the corresponding C–C atom distances computed at a force of 2.0 nN. The potential activation energy of the *syn*-CPDs reaches 0 kcal mol^−1^, at *F*_max_ = 2.9 nN (*cis*) and *F*_max_ = 3.0 nN (*trans*), which is in line with CoGEF being able to detect mechanochemical activatability. For the *anti*-CPDs, there is only one transition state detectable along the total pathway from CPD to the diene products. This is reflected in the qualitatively different C–C distances of the transition state structures. The barrier heights for the second C–C bond breaking of the *syn*-CPDs can be found in the SI.

To further elucidate the mechanochemical ring opening of the four isomeric CPDs, the transition state structures and reactant structures were localized with the FM-PES method (Fig. 9). It can be seen that the force transduction is stronger for the *syn*-CPDs compared to the *anti*-CPDs as the potential energy barriers decrease more strongly with increasing force for the *syn*-CPDs. We attribute this to the more direct force transduction to the first C–C bond of the *syn*-CPD, making them a good candidate for mechanochemical activation. For these *syn*-CPD motifs, the barrier of the second bond breaking is below 3.0 kcal mol^−1^ throughout, which will lead to an immediate, second C–C bond breaking once the barrier of the first C–C bond breaking is overcome. The difference in the 
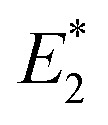
 values computed with the CoGEF method for *cis*- and *trans*-isomers stems from twisting of the molecular structure, which leads to a larger *F*_max_, for the *trans-*isomer. The FM-PES computations indeed show existence of a minimum structure related to the twisted biradical intermediate up to 5.0 nN. However, with an associated barrier for the second C–C bond breaking of below 2.0 kcal mol^−1^ throughout, this biradical intermediate is not persistent, but will open to the products immediately once formed. In contrast, the diagonal configuration of the cyclobutane unit of the *anti*-CPDs leads to a smaller force transduction to the mechanophore. As a consequence, the barrier heights stay above 35 kcal mol^−1^ in the range of mechanochemical relevance (4 nN). Furthermore, only one single transition state structure involving breaking of both C–C bonds could be located for the *anti*-CPDs (Fig. 9, lower panel) and no biradical intermediate can be localized. We collected the most important parameters from CoGEF and FM-PES computations in Table 3. Altogether, both *syn*-CPDs can be regarded as functional mechanophores and, in agreement with the CoGEF computations, a lower mechanochemical reactivity can be expected for the *anti*-CPDs.

**Table 3:** The energy and force required for the rupture of the four isomeric structures and their mechanochemical reactivity. Values for 
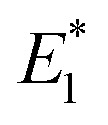
, 
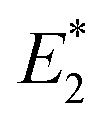
 (obtained from CoGEF computations), and the barriers at 2.0 nN are in kcal mol^−1^. The rupture forces *F*_max_ were determined by the CoGEF method

Derivative	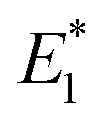	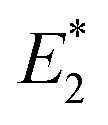	*F*_max,1_	*F*_max,2_	Barrier at 2.0 nN	Mechanochemical reactivity
*cis*–*syn*	65.95	66.92	2.9 nN	1.2 nN	7.41^a^	Higher
*trans*–*syn*	75.34	186.12	3.2 nN	5.0 nN	8.34^a^	Higher
*cis*–*anti*	—	176.16		6.0 nN	45.73	Lower
*trans*–*anti*	—	194.81		6.0 nN	44.63	Lower

a Barrier for the first C–C bond breaking.

## 3 Conclusions

In summary, we introduced CPDs as a novel class of mechanophores. Using complementary techniques including GPC, NMR, and computational simulations, we demonstrated that CPDs undergo cycloelimination under 20 kHz ultrasound irradiation, restoring the original thymidine structure in the sonication products. We quantified the rate constants of this mechanochemical scission across varying polymer molar masses and ultrasound intensities, further characterizing their force-responsive behavior. Ultrasound-induced bond scission of the different isomers suggested a reactivity difference between the *cis*–*syn* and the *trans*–*anti* isomers. Further, computational investigations using force application by CoGEF and FM-PES allowed inferring that *syn*-isomers are mechanochemically more active, while *anti*-isomers are less sensitive to force-induced cleavage. Together, these experimental and computational findings establish CPDs as nucleobase- and nucleoside-derived mechanophores, laying a foundation for their future application in biologically relevant systems and biohybrid materials.

## Author contributions

Conceptualization, X. G., G. C., J. M., R. G., and A. H.; methodology, X. G., J. M., R. G., and A. H.; investigation, X. G., F. J. U., M. L.-B., R. L., J. F., and K. S.; writing – original draft, X. G., G. C., F. J. U., M. L.-B., R. L., and J. M.; writing – review and editing, X. G., G. C., F. J. U., M. L.-B., R. L., J. M.; R. G., and A. H.; funding acquisition, J. M., A. H.; resources, J. M., A. H.; supervision, G. C., J. M., R. G., and A. H.

## Conflicts of interest

There are no conflicts of interest to declare.

## Supplementary Material

SC-017-D6SC02165D-s001

## Data Availability

The data supporting this article have been included as part of the supplementary information (SI). Supplementary information (SI) is available. See DOI: https://doi.org/10.1039/d6sc02165d.
