# Supplementary material for: The mechanochemical activation of a pyrimidine dimer
Source: Chem Sci. 2026 Apr 17;17(21):10589–99. doi: 10.1039/d6sc02165d (PMC13088988; doi:10.1039/d6sc02165d)
Supplement: SC-017-D6SC02165D-s001 [file SC-017-D6SC02165D-s001.pdf]

Supplementary Information (SI) for Chemical Science.
This journal is © The Royal Society of Chemistry 2026

Supporting Information for

The Mechanochemical Activation of a Pyrimidine Dimer

Xiang Gao[1,2], Gurudas Chakraborty[2,*], Felix Joel Urhahne[3], Marcus Lantzius-Beninga[1,2], Regina Lennarz[3], Jilin Fan[1,2], Kara Stappert[1], Jan Meisner[3], Robert Göstl[2,4], and Andreas Herrmann[1,2,*]

[1]Institute of Technical and Macromolecular Chemistry, RWTH Aachen University, Worringerweg 2, 52074 Aachen, Germany

[2]DWI – Leibniz-Institute for Interactive Materials, Forckenbeckstraße 50, 52056 Aachen, Germany

[3]Institute for Physical Chemistry, Heinrich Heine University Düsseldorf, Universitätsstraße 1, 40225 Düsseldorf, Germany

[4]Department of Chemistry and Biology, University of Wuppertal, Gaußstraße 20, 42119 Wuppertal, Germany

*To whom correspondence should be addressed.

E-Mail: chakraborty@dwi.rwth-aachen.de, herrmann@dwi.rwth-aachen.de

Table of Contents

I. Materials S2
II. Methods S2
III. Synthetic Details S3
IV. Controlled Radical Polymerization S8
V. Sonication Experiments S11
VI.Study on the individual isomers and the control PMA S12
VII. Computational Details S18
VIII. Potential energies of the stationary points and activation energies of the CPD mechanophores S19
IX. References S23

Experimental Section

Materials

All chemical reagents were of analytical grade and used without further purification: Thymidine (98%, abcr GmbH), 2-Bromoisobutyryl bromide (>98%, TCI), 4-Dimethylaminopyridine (>99%, TCI), Methyl acrylate (99%, Sigma Aldrich). Prior to the polymerization, methyl acrylate was filtered over basic Al_2O_3. The other chemicals were purchased from Sigma Aldrich unless otherwise noted.

Methods

NMR spectra were recorded in DMSO-d_6, D_2O or MeOH-d_4 on a 400 MHz Bruker Avance 300 or 400 spectrometer (^{13}C: 75 or 101 MHz). Chemical shifts are reported in δ units using residual protonated solvent signals as internal standard[1] (^1H: DMSO-d_6 (δ_H = 2.50 ppm), D_2O (δ_H = 4.79 ppm) or MeOH-d_4 (δ_H = 3.31 ppm); ^{13}C: MeOH-d_4 (δ_C = 49.00 ppm)). The following abbreviations were used throughout: s = singlet, d = doublet, t = triplet, q = quartet, sept. = septet, dd = doublet of doublet etc., m = multiplet. Coupling constants (J) are given in Hz and refer to the given H,H-couplings.

TLC was performed on Merck TLC Silica gel 60 F254 TLC plates with a fluorescent indicator employing a 254 nm or 365 nm UV-lamp for visualization. Silica gel for chromatography (40-63 µm) was used for flash column chromatography.

High-resolution ESI-TOF mass spectrometry was performed on a micrOTOF-QII instrument from Bruker Daltonik.

Gel permeation chromatography (GPC/SEC) with THF (HPLC grade, VWR) as eluent was performed using a HPLC pump (PU-2080plus, Jasco) equipped with a refractive index detector (RI-2031plus, Jasco). The sample solvent contained 250 $mg \cdot mL^{-1}$ 3,5-di-t-4-butylhydroxytoluene (BHT, ≥99%, Fluka) as internal standard. One pre-column (8×50 mm) and four SDplus gel columns (8×300 mm, SDplus, MZ Analysentechnik) were applied at a flow rate of 1.0 $mL \cdot min^{-1}$ at 20 °C. The diameter of the gel particles was 5 µm, the nominal pore widths were 50, 10^2, 10^3, and 10^4 Å. Calibration was achieved using narrowly distributed poly(methyl methacrylate) standards (Polymer Standards Service). Molecular weights (*Mn*,

SEC and Mw, SEC) and molar mass distributions (*Mw*/*Mn*) were calculated by using the PSS WinGPC UniChrom software (Version 8.1.1).

Sonochemical irradiation experiments were carried out with a VibraCellTM ultrasonic processor VCX500 (20 kHz) purchased from Sonics & Materials in a Suslick vessel purchased from Sonics & Materials under inert atmosphere while cooling with an ice bath. The A12627PRB20 probe was used for all sonication experiments.

The photoreactions were exposed to UV-B irradiation using four 36 W 310 nm wavelength Hg lamps purchased from Philips company.

Synthetic Details

Cyclobutane thymidine dimer **1** (CPD) [2]

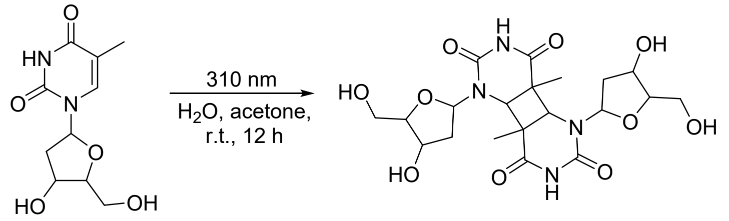

Thymidine (484 mg, 2 mmol, 1 eq.) was dissolved in 100 mL acetone and water (1:2). Irradiation was performed under four 36 W 310 nm UV-B light. After 12 hours, the solvent was removed, and the crude product was purified by column chromatography on silica gel (methanol : dichloromethane = 1:6) to obtain a white solid **1** (164 mg, 34% yield).

^{1}H-NMR (400 MHz, D_2O) δ 6.33-5.36(m, 2H), 4.79-3.62(m, 10H), 2.62-2.07(m, 4H), 1.54-1.36(m, 6H). See **Figure S1**

^{13}C-NMR (101 MHz, D_2O) δ 174.31, 173.91, 173.07, 172.39, 172.17, 153.70, 153.13, 152.65, 152.51, 152.10, 151.82, 88.77, 86.73, 86.39, 86.26, 86.00, 85.83, 85.65, 85.52, 85.43, 85.38, 85.00, 84.08, 83.29, 72.22, 71.66, 71.35, 71.21, 70.81, 70.60, 62.20, 61.70, 61.67, 60.01, 59.36, 57.03, 56.71, 55.27, 49.14, 49.06, 48.88, 47.83, 47.38, 47.13, 46.91, 46.37, 38.93, 37.51, 37.32, 36.89, 36.37, 21.21, 18.55, 18.31, 17.42, 17.13, 17.02, 16.99. See **Figure S2**

MS(ESI+): m/z Calcd for $C_{20}H_{28}N_4O_{10}$ $[M+Na]^+$ 507.1698, Found for $[M+Na]^+$ 507.1662.
See **Figure S3**

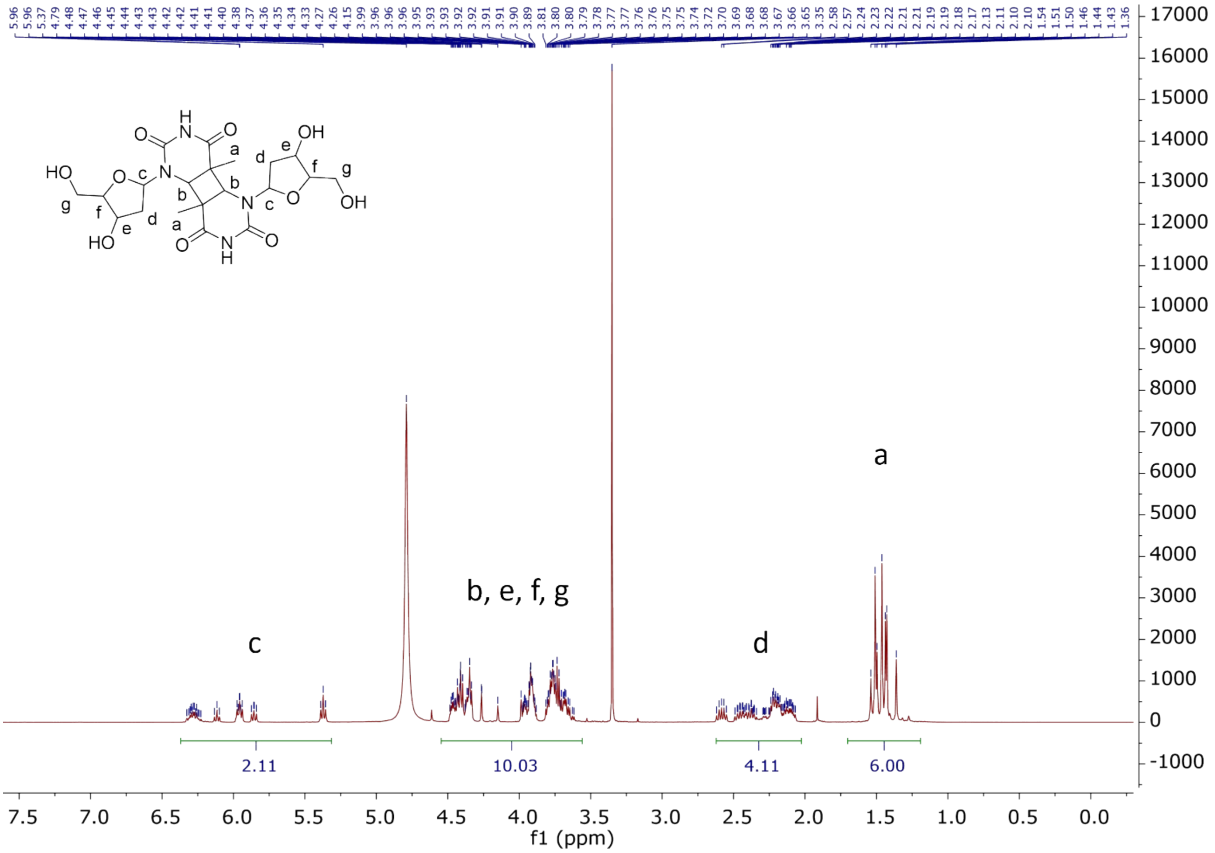

Figure S1. ^{1}H-NMR spectrum of cyclobutane thymidine dimer **1**.

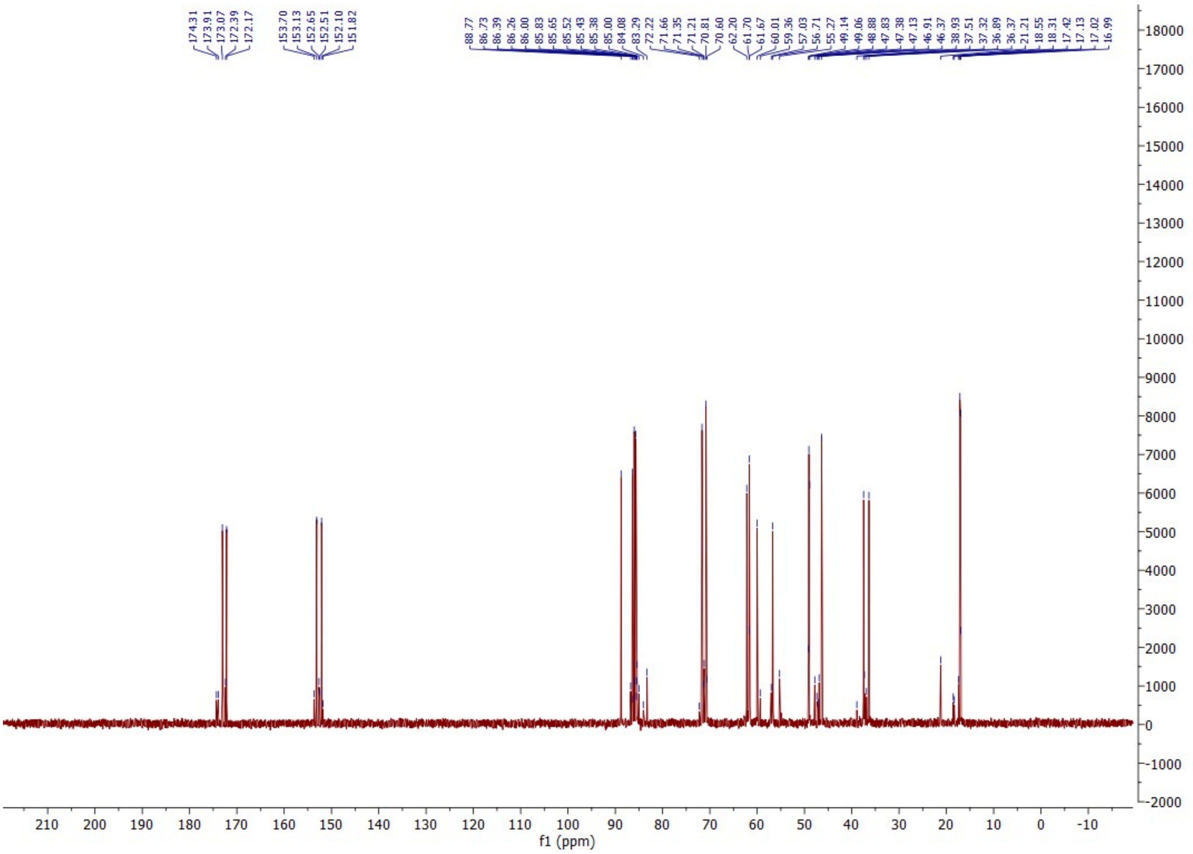

Figure S2. ^{13}C-NMR spectrum of cyclobutane thymidine dimer **1**.

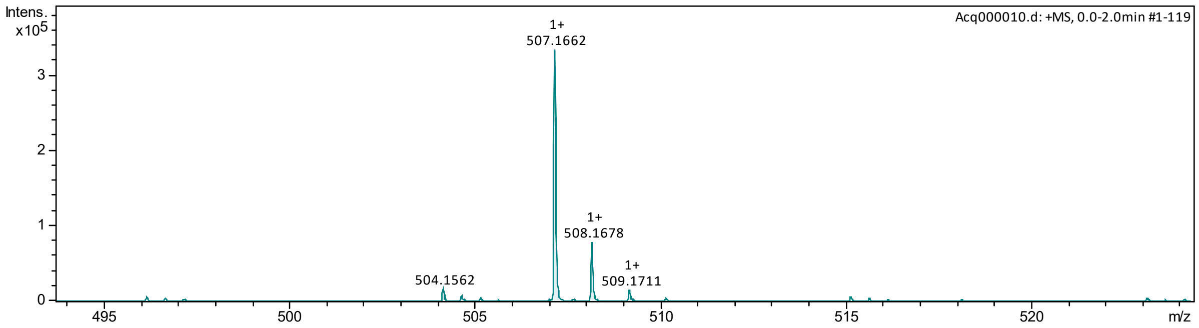

Figure S3. ESI+ mass spectrum of cyclobutane thymidine dimer **1**.

Cyclobutane thymidine dimer initiator **2** [3]

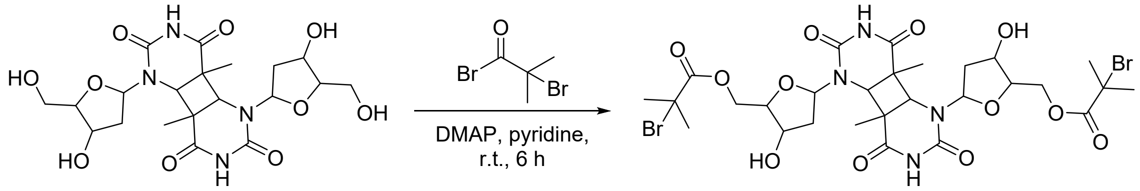

1 (242 mg, 0.5 mmol, 1 eq.) and 4-dimethylaminopyridine (5 mg) were dissolved in 5 mL pyridine. Next, pyridine was removed under reduced pressure and this process was repeated three times. Under nitrogen atmosphere, 10 mL anhydrous pyridine was added and 2-bromoisobutyryl bromide (1.35 mL, 1.1 mmol, 2.2 eq.) was added dropwise. After 6 h reaction at room temperature, the reaction was stopped with the addition of a small amount of methanol. The solvent was removed, and the crude product was purified by column chromatography on silica gel (eluent gradient, 30%-100% ethylactate in cyclohexane) to obtain a milky white solid **2** (218 mg, 56% yield).

^{1}H-NMR (400 MHz, MeOH-d$_4$) δ 6.31-5.29(m, 2H), 4.47-3.77(m, 10H), 2.70-2.01(m, 4H), 1.96-1.90(m, 12H), 1.52-1.29(m, 6H). See **Figure S4**

^{13}C-NMR (101 MHz, MeOH-d$_4$) δ 171.61, 171.45, 171.40, 171.38, 171.35, 171.01, 170.96, 170.92, 170.80, 170.20, 152.28, 151.86, 151.16, 151.10, 89.44, 88.15, 87.67, 86.49, 83.86, 83.24, 81.35, 80.63, 76.58, 75.93, 71.32, 70.97, 65.63, 65.19, 65.12, 60.71, 59.06, 58.16, 56.82, 56.07, 56.03, 56.01, 55.99, 55.93, 55.46, 55.42, 49.40, 48.31, 48.10, 47.89, 47.67, 47.55, 47.46, 47.25, 47.03, 46.98, 45.28, 37.96, 36.79, 34.78, 33.77, 29.89, 29.85, 29.83, 29.79, 29.59, 29.56, 29.53, 29.46, 17.39, 17.29, 17.26. See **Figure S5**

MS(ESI$^+$): m/z Calcd for $C_{28}H_{38}Br_2N_4O_{12}$ $[M+Na]^+$ 805.0751, Found for $[M+Na]^+$ 805.0670. See **Figure S6**

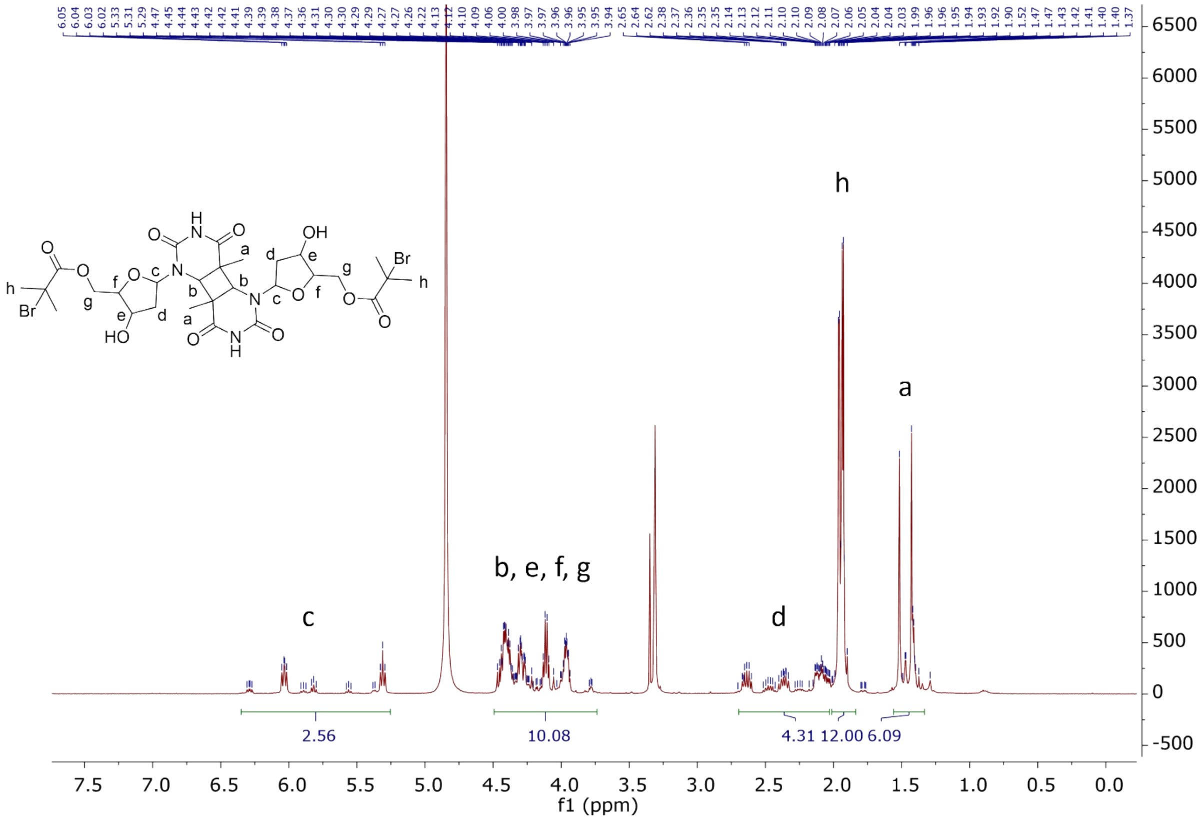

Figure S4. ^{1}H-NMR spectrum of cyclobutane thymidine dimer initiator **2**.

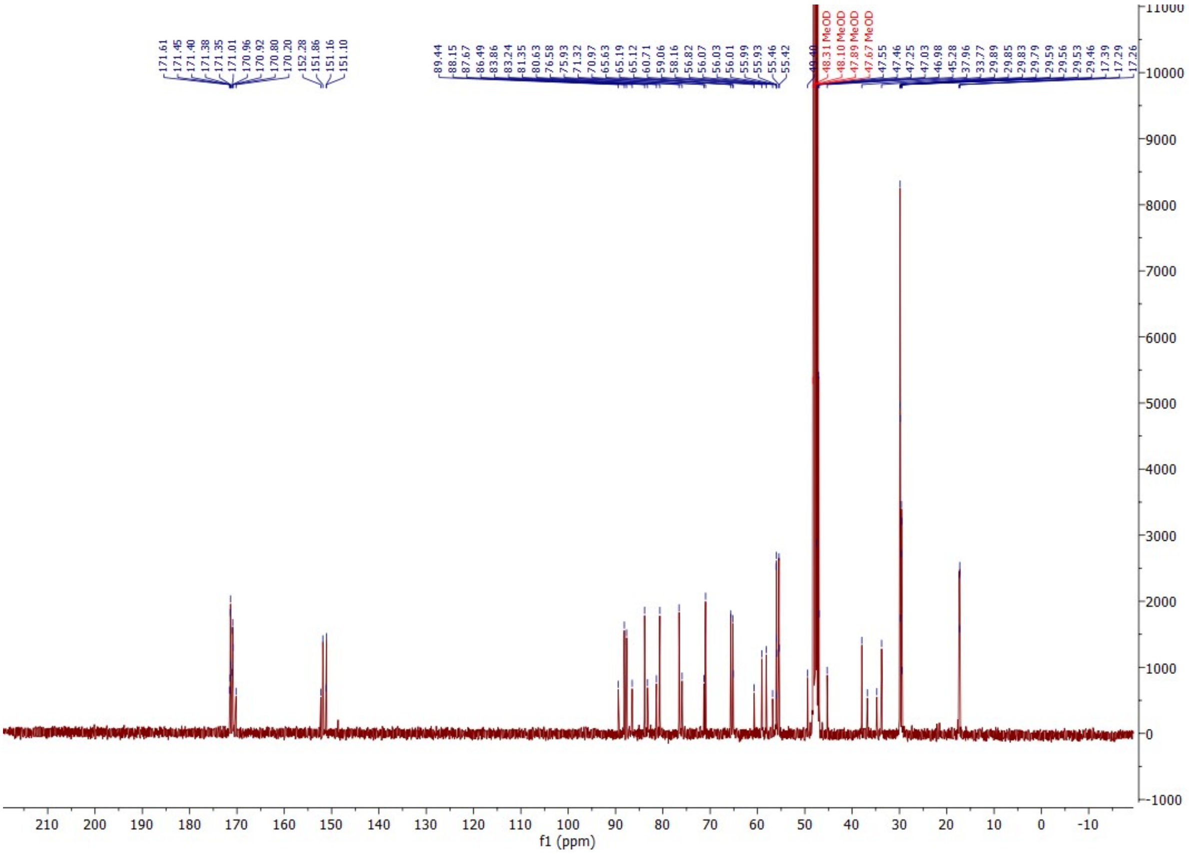

Figure S5. ^{13}C-NMR spectrum of cyclobutane thymidine dimer initiator **2**.

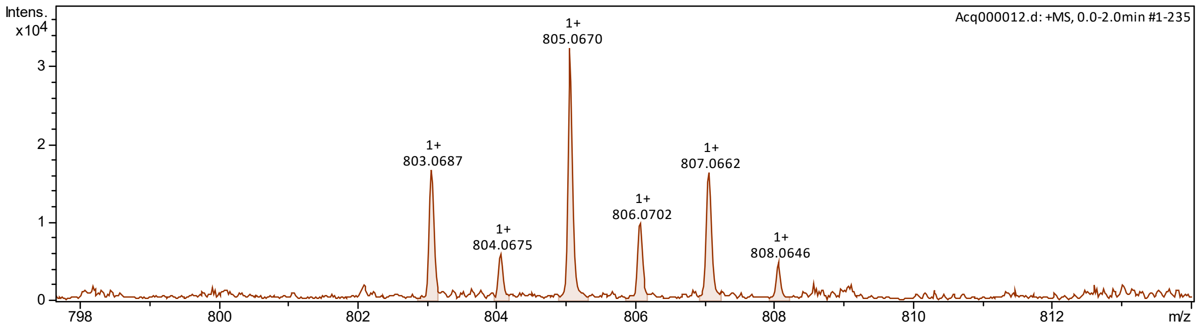

Figure S6. ESI+ mass spectrum of cyclobutane thymidine dimer initiator **2**.

To verify the selective esterification of the primary alcohols of the CPD, we recorded HR-ESI MS and did not detect any masses corresponding to a tri- or quadruple-functionalized initiator (**Fig. S7**). Additionally, we applied the same esterification reaction conditions to non-dimerized thymidine as a model reaction to examine the amount of secondary alcohol esterification by NMR spectroscopy (**Figs. S8-S9**). The ^{1}H NMR spectrum shows the formation of only 17% (0.22/(1.08+0.22)) secondary alcohol esterification (signal at ca. 5.75 ppm) under the applied conditions, demonstrating the selective transformation. The remaining side product was removed during the photodimer initiator isolation by column chromatography.

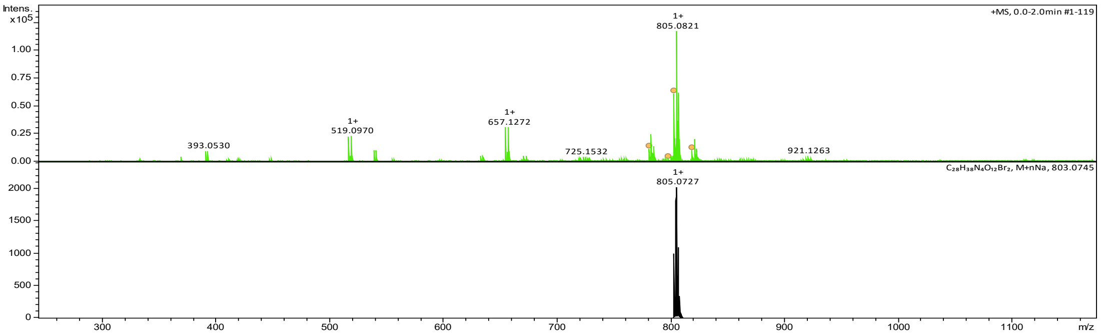

Figure S7. Full HR-ESI MS spectrogram of cyclobutane thymidine dimer initiator **2**.

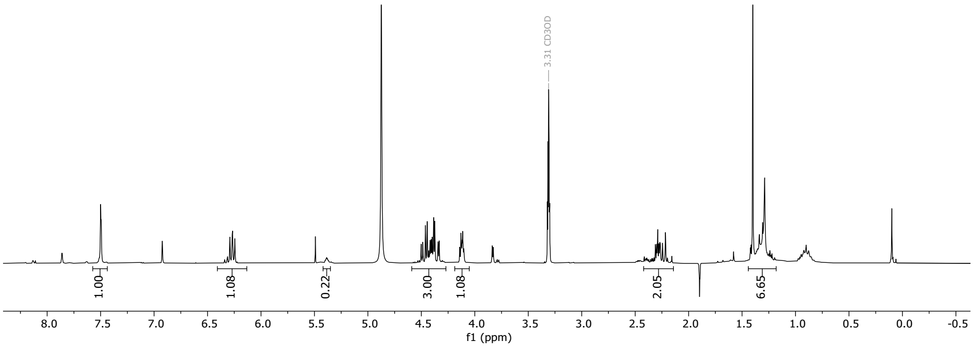

Figure S8. ^{1}H NMR spectrum (water suppression NMR experiment noesygppr 1d) of thymidine initiator.

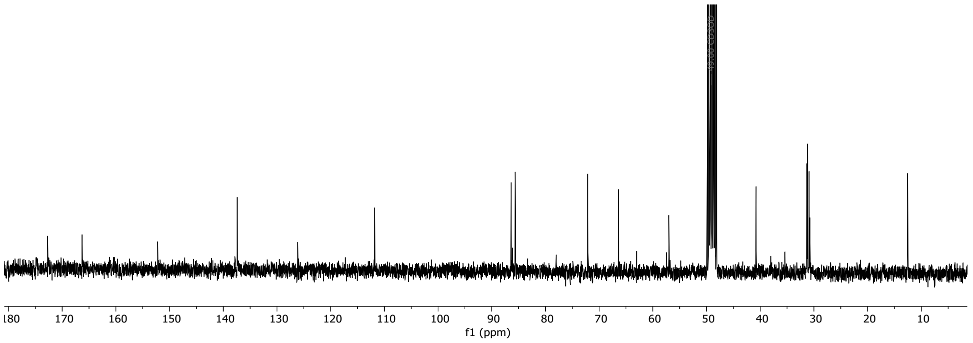

Figure S9. ^{13}C NMR spectrum of thymidine initiator.

Controlled Radical Polymerization [4]

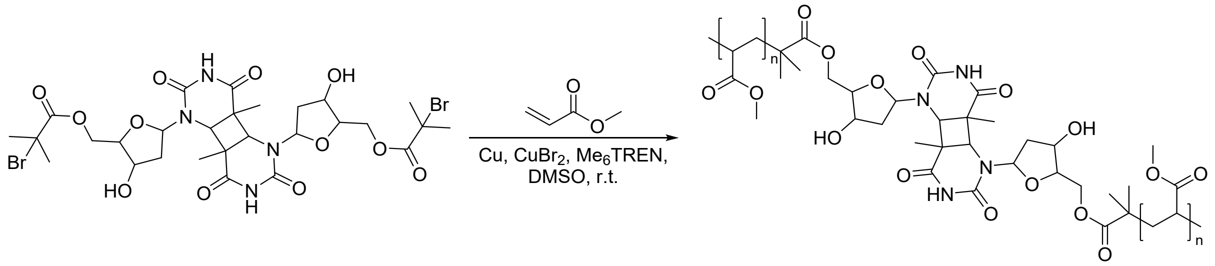

Before the reaction, a $CuBr_2$ solution A in DMSO (11.6 mg/mL, 0.05 mmol/mL) and a Me_6-TREN solution B in DMSO (36.8 mg/mL, 0.16 mmol/mL) were prepared, and the copper wire (4 cm) was activated by soaking it in diluted hydrochloric acid for 30 minutes before washing it with water and acetone. In a Schlenk flask, mechanophore initiator **2** (20 mg, 0.025 mmol, 1 eq.), the $CuBr_2$ solution A (1.25 µmol, 0.05 eq.), the Me_6-TREN solution B (4 µmol, 0.16 eq.), filtered monomer methyl acrylate (600-1200 eq.), and dry DMSO (5 mL) were degassed by freeze-pump-thaw cycling. The polymerization commenced after dropping the copper-wire into the degassed mixture. The reaction mixture was stirred at room temperature for the indicated reaction time. Afterwards, the mixture was diluted with THF and filtered over basic Al_2O_3. The solvent was removed under vacuum, followed by precipitation in cold MeOH to yield the desired poly(methyl acrylate) (PMA). (**Table S1**)

Table S1. Reaction conditions of polymer and molecular weight distribution obtained by GPC.

PMA	Monomer	Time	M_n	$Đ_M$	Figure
1	1500 eq.	5 h	104 kDa	1.18	S10
2	1200 eq.	5 h	71 kDa	1.27	S11
3	600 eq.	5 h	49 kDa	1.24	S12
4	600 eq.	3 h	33 kDa	1.31	S13

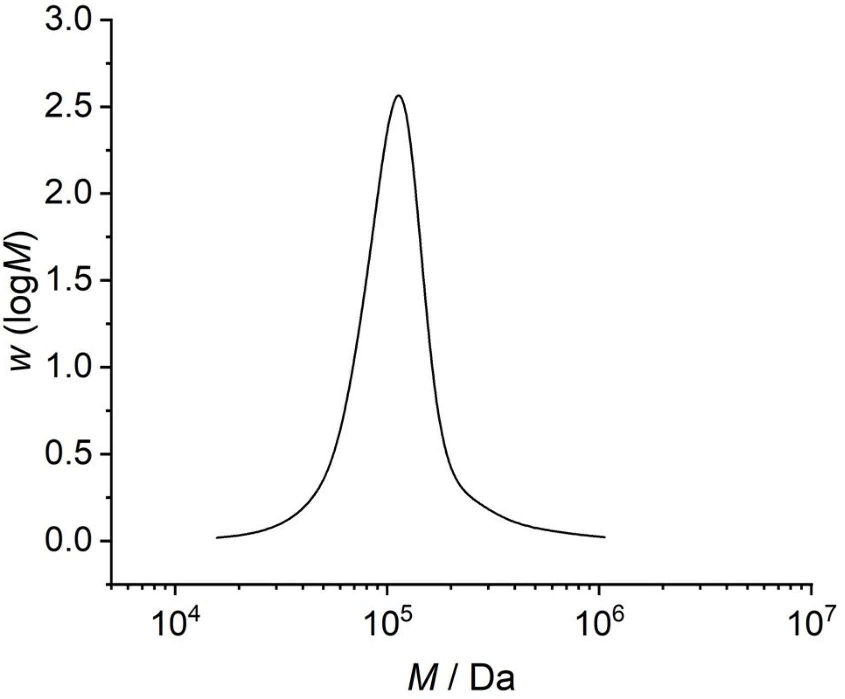

Figure S10. Differential molar mass distribution of GPC RI chromatogram of **PMA 1**.

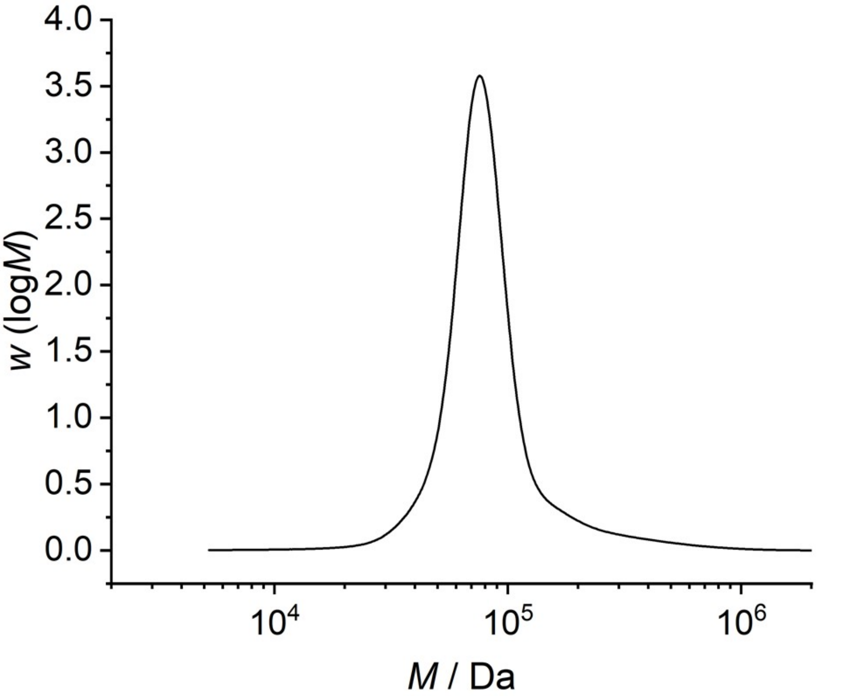

Figure S11. Differential molar mass distribution of GPC RI chromatogram of **PMA 2**.

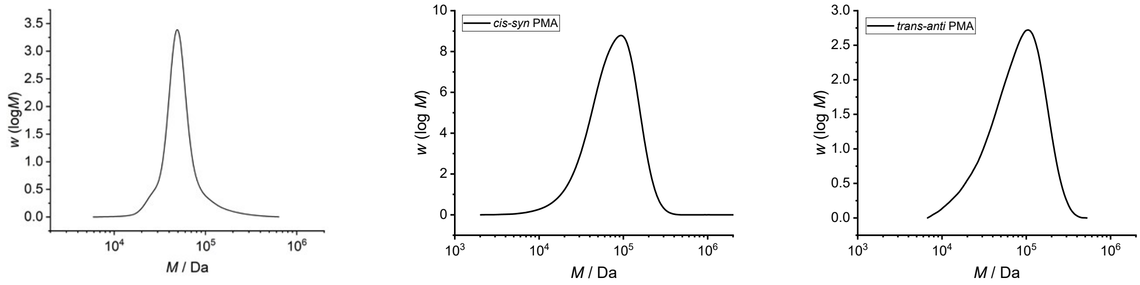

Figure S12. Differential molar mass distribution of GPC RI chromatogram of **PMA 3**, *cis-syn* PMA, and *trans-anti* PMA (from left to right).

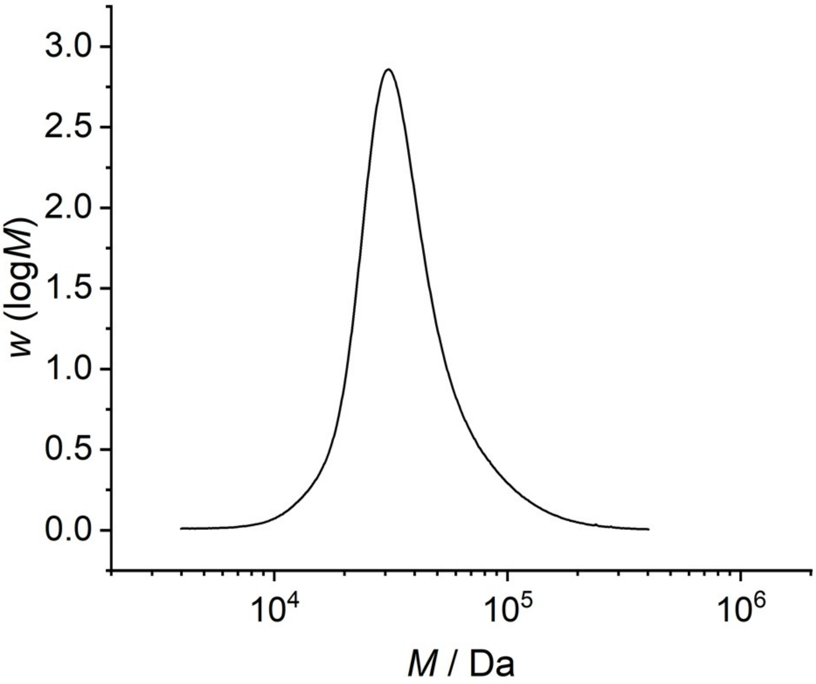

Figure S13. Differential molar mass distribution of GPC RI chromatogram of **PMA 4**.

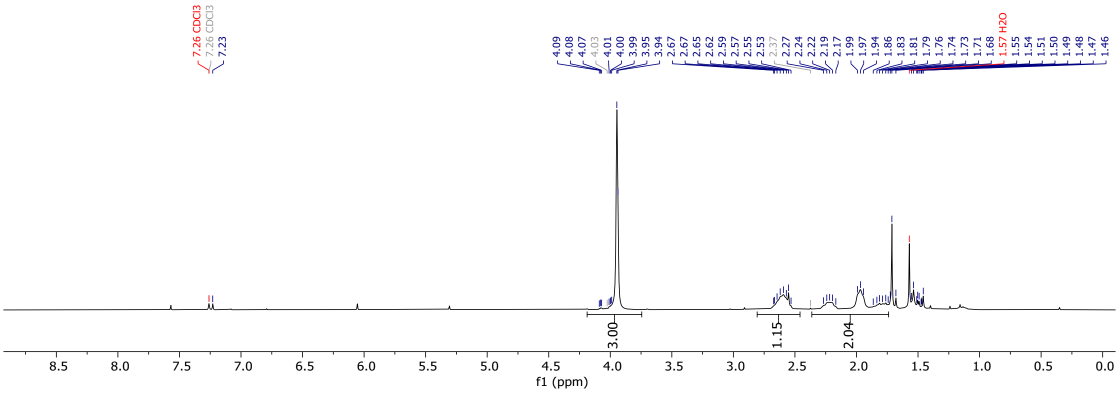

Figure S14. Representative ^1H NMR spectrum of PMA polymer with CPD incorporated.

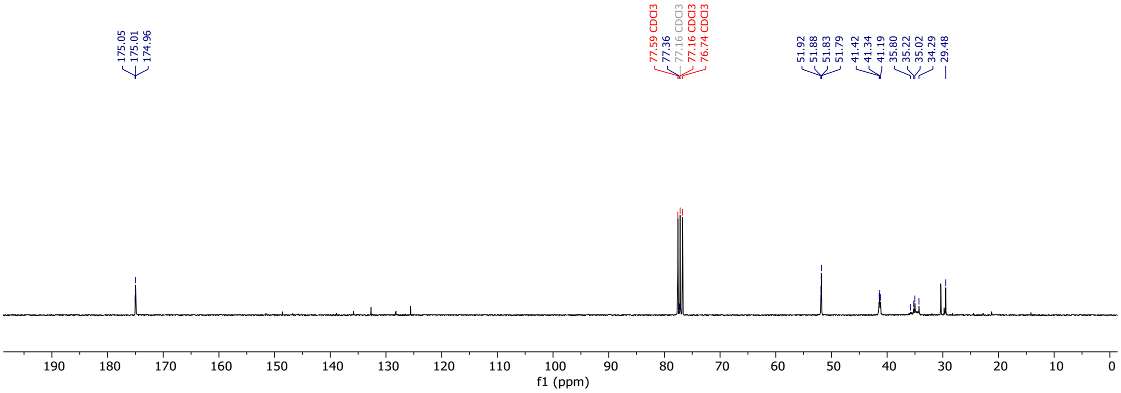

Figure S15. Representative ^{13}C NMR spectrum of PMA polymer with CPD incorporated. The signal at ca. 30 ppm is caused by residual acetone.

Sonication experiments

100 mg **PMA 1-4**, *cis-syn* PMA/*trans-anti* PMA, or the control PMA were dissolved in 10 mL degassed DMSO and injected into a cooled Suslick cell (immersed in an ice-water bath). The mixture was exposed to 20 kHz sonication via a 3mm probe (A12627PRB20). Pulsed sonication (2 s on, 1 s off) was used. Further details are shown in the **Table S2**.

Table S2. Sonication reaction conditions

Amplitude setting /%	Total input energy per 600s /J	Input work in solution $W_{solution}$ / W	Input work in air W_{air}/W	Ultrasound intensity I_P /W·cm^{-2}
80	4788	7.98	7	14
60	2831	4.72	4	10
40	1447	2.41	2	6
20	130	0.22	0	3

In above table, total input energy was read directly from the machine, input wattage in air was obtained by the instrument instruction manual, the sonication intensity is calculated by the following **Equation S1**:

$$I_P = \frac{Wsolution - Wair}{\pi r2}$$

Equation S1

I_P : Sonication intensity

$W_{solution}$: Input work in solution

W_{air} : Input work in air

r : Radius of the probe

Study on the individual isomers and the control PMA

The CPD isomers were isolated by preparative HPLC on a C18 column in aqueous ammonium acetate solution containing methanol. The isolated isomers were identified according to literature[1] and by ^{1}H, ^{13}C, and 2D NMR experiments (**Figs. S16-S19**). Their mechanochemical activation upon ultrasonication was studied using PMA polymers of comparable molecular weight, and two different methods to determine the scission rate were applied (**Figs. S20-S21**). As a control experiment to exclude substantial random scission of the polymer chains, we prepared a non-functionalized PMA (without a mechanophore) with a comparable molecular weight and subjected it to sonication. A substantially lower bond scission was observed, confirming the mechanochemical activation of CPD as the main scission mechanism (**Figs. S20-22**).

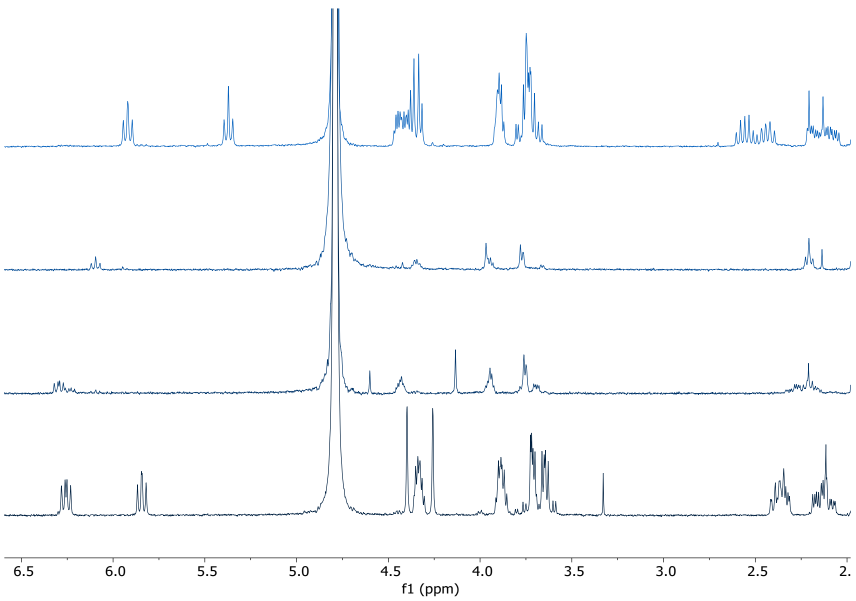

Figure S16. ^{1}H NMR spectra of the isolated isomers of thymidine photodimer. From top to bottom: *cis-syn*, *cis-anti* (−), *cis-anti* (+), *trans-anti*. The *trans-syn* isomer is only formed in minor portion (<10%) and the isolated amount did not allow a characterization by NMR.

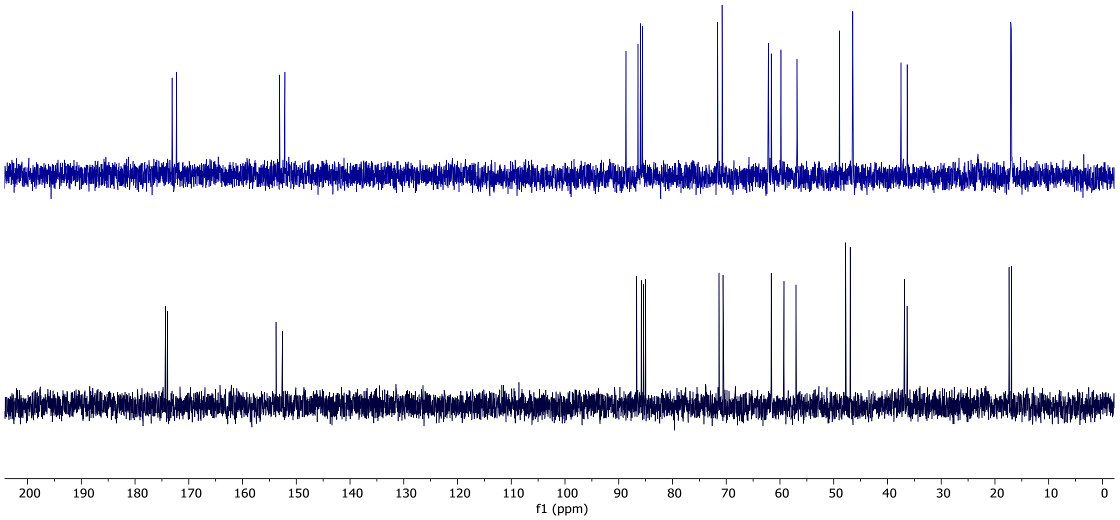

Figure S17. ^{13}C NMR spectra of the isolated isomers of thymidine photodimer. From top to bottom: *cis-syn*, *trans-anti*.

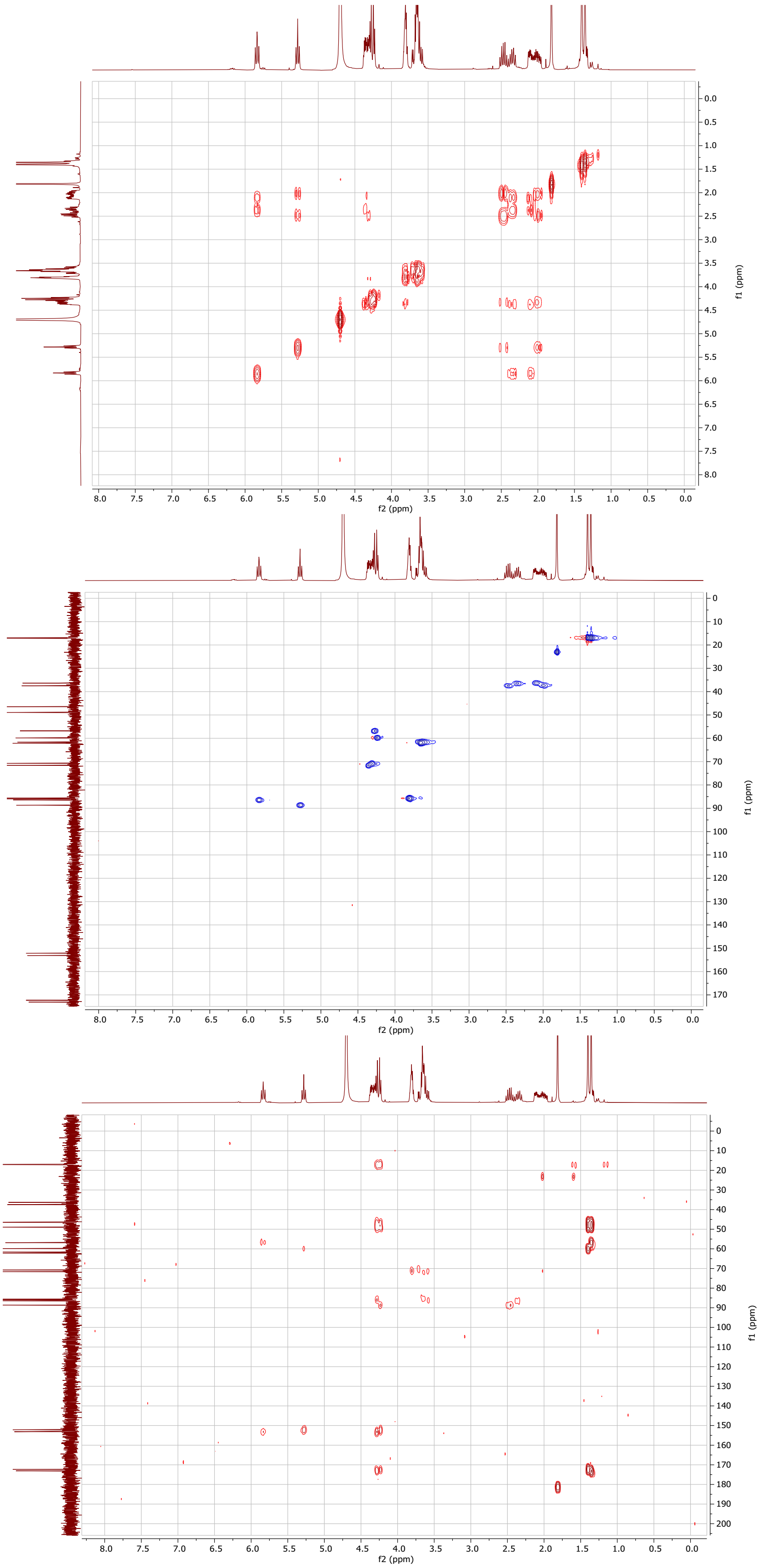

Figure S18. 2D NMR spectra of the isolated *cis-syn* isomer of the thymidine photodimer. From top to bottom: ^{1}H-^{1}H COSY, ^{1}H-^{13}C HSQC, ^{1}H-^{13}C HMBC.

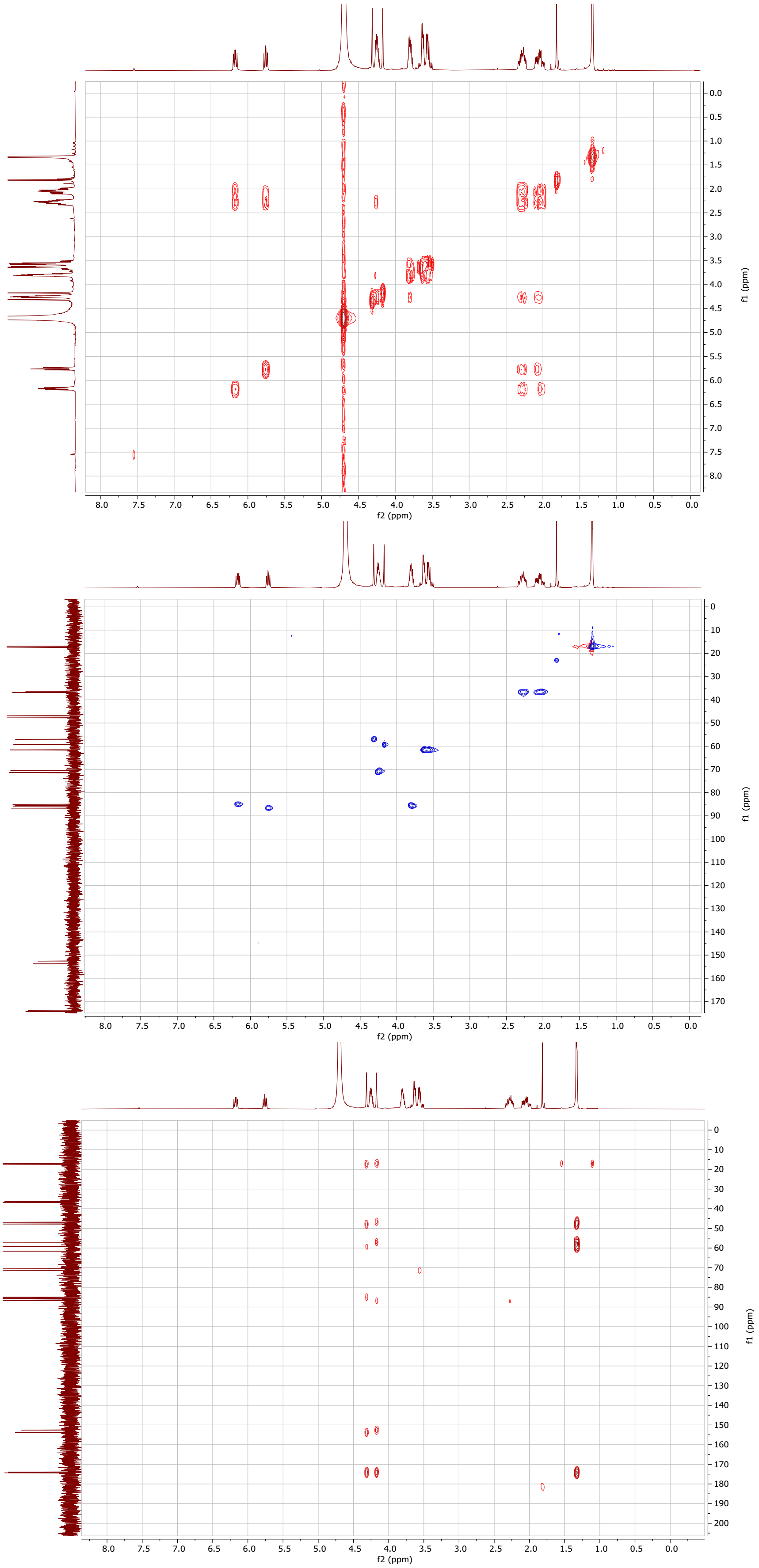

Figure S19. 2D NMR spectra of the isolated *trans-anti* isomer of the thymidine photodimer. From top to bottom: ^{1}H-^{1}H COSY, ^{1}H-^{13}C HSQC, ^{1}H-^{13}C HMBC.

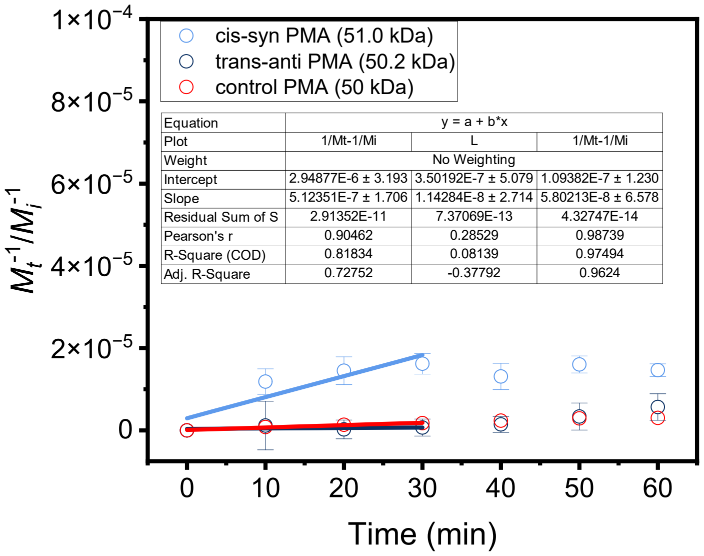

Figure S20. Plot of M_t^{-1}-M_i^{-1} vs. t for PMA containing the *cis-syn* or *trans-anti* isomer of the thymidine photodimer or no mechanophore (control PMA). Error bars represent standard deviation derived from independent experiments.

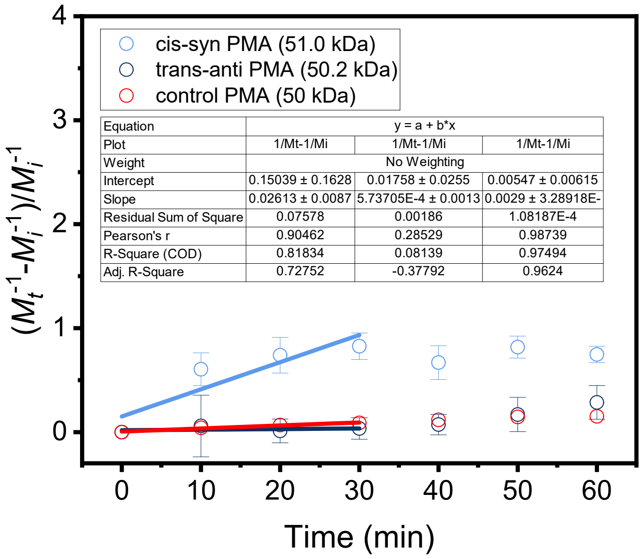

Figure S21. Plot of $(M_t^{-1}$-$M_i^{-1})/M_i^{-1}$ vs. t for PMA containing the *cis-syn* or *trans-anti* isomer of the thymidine photodimer or no mechanophore (control PMA). Error bars represent standard deviation derived from independent experiments.

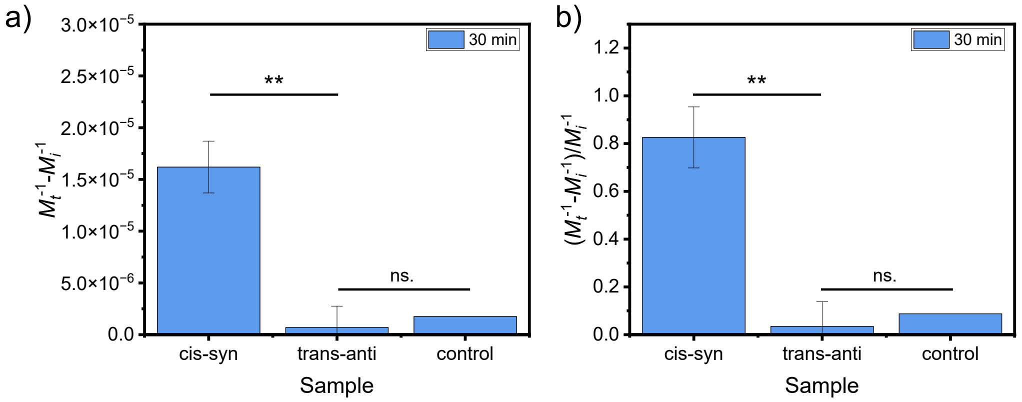

Figure S22. Fraction of scission M_t^{-1}-M_i^{-1} (a) and (M_t^{-1}-M_i^{-1})/M_i^{-1} (b) for *cis-syn*, *trans-anti*, and the control PMA after 30 minutes ultrasonication. ** indicates $p < 0.01$.

Computational Details

Initial structures were derived using ChemDraw and Avogadro.[6] Calculations were performed at the level of 'broken symmetry' DFT, employing the unrestricted B3LYP functional[7-10] and the 6-31G* basis set.[11-13] The threshold for SCF convergence was set to $1 \cdot 10^{-6}$ Hartree.

Constrained Geometries Simulate External Force (CoGEF) calculations[14, 15] were performed using TeraChem,[16, 17] with a tolerance value for the gradient of $4.5 \cdot 10^{-4}$. An optimized structure for the reactant without the influence of external force was selected as the initial structure for the constrained optimizations. During the CoGEF simulations, the distance between the attachment points where the force is applied was increased in increments of 0.1 Å. The maximum force F_{max} before rupture of the mechanophore was obtained by analyzing the gradient of the energy-displacement curve. Typically, F_{max} corresponds to the energy point just before a discontinuity in this curve, and its value is calculated from the gradient between the two data points immediately preceding the discontinuity.

Stationary points were located on the force-modified potential energy surface (FMPES).[18] Respective calculations were performed using DL-FIND,[19] which is interfaced to TeraChem *via* ChemShell.[20] A gradient tolerance of $4.5 \cdot 10^{-5}$ was used as the convergence criterion for optimization. It was assumed that the force remained constant throughout the entire reaction time scale. Therefore, the external force could be calculated as the sum of the electronic *ab initio* potential and a mechanical force term, $-F \cdot \Delta x$, where F is the force and Δx is the distance between the two atoms to which the force is applied. The force was varied between 0.0 nN and 6.0 nN in steps of 0.5 nN, using steps of 0.1 nN when necessary. To confirm that the optimized geometries were minima, the eigenvalues of the Hessian matrix were analyzed, since minima are characterized by having only positive eigenvalues. Transition state structures were optimized using the dimer method.[21, 22] The stationary points identified as transition states were also confirmed by examining the Hessian, which exhibits exactly one negative eigenvalue for first-order saddle points.

Potential energies of the stationary points and activation energies of the CPD mechanophores

Table S3. Calculated absolute potential energies V in atomic units for the reactant and transition state and the respective activation energy V_A in kcal/mol for the first step of the ring opening reaction of *cis-syn* CPD.

	Reactant	Transition State	
Force [nN]	V[a.u.]	V[a.u.]	Potential activation energy V_A [kcal/mol]
0.0	-2055.7246	-2055.6485	47.74
0.5	-2055.9292	-2055.8761	33.33
1.0	-2056.1479	-2056.1131	21.82
1.5	-2056.3762	-2056.3538	14.06
2.0	-2056.6091	-2056.5973	7.41
2.5	-2056.8464	-2056.8428	2.28
2.9	-2057.0396	-2057.0393	0.19

Table S4. Calculated absolute potential energies V in atomic units for the reactant and transition state and the respective activation energy V_A in kcal/mol for the first reaction step of the ring opening reaction of *trans-syn* CPD.

	Reactant	Transition State	
Force [nN]	V[a.u.]	V[a.u.]	Potential activation energy V_A [kcal/mol]
0.0	-2055.7358	-2055.6462	56.70
0.5	-2055.9276	-2055.8747	33.19
1.0	-2056.1509	-2056.1134	23.51
1.5	-2056.3788	-2056.3554	14.69
2.0	-2056.6134	-2056.6001	8.34
2.5	-2056.8520	-2056.8457	3.97
3.0	-2057.0945	-2057.0923	1.42

Table S5. Calculated absolute potential energies V in atomic units for the reactant and transition state and the respective activation energy V_A in kcal/mol for the ring opening reaction of the *cis-anti* CPD.

	Reactant	Transition State	
Force [nN]	V[a.u.]	V[a.u.]	Potential activation energy V_A [kcal/mol]
0.0	-2055.7332	-2055.6508	51.70
0.5	-2055.9640	-2055.8840	50.20
1.0	-2056.2053	-2056.1276	48.79
1.5	-2056.4500	-2056.3745	47.43
2.0	-2056.6976	-2056.6247	45.73
2.5	-2056.9477	-2056.8781	43.70
3.0	-2057.2006	-2057.1349	41.22
3.5	-2057.4563	-2057.3949	38.50
4.0	-2057.7184	-2057.6584	37.65
4.5	-2057.9805	-2057.9257	34.37
5.0	-2058.2461	-2058.1964	31.21
5.5	-2058.5158	-2058.4706	28.38
5.9	-2058.7353	-2058.6939	25.96

Table S6. Calculated absolute potential energies V in atomic units for the reactant and transition state and the respective activation energy V_A in kcal/mol for the ring opening reaction of the *trans-anti* CPD.

	Reactant	Transition State	
Force [nN]	V[a.u.]	V[a.u.]	Potential activation energy V_A [kcal/mol]
0.0	-2055.7377	-2055.6602	48.66
0.5	-2055.9518	-2055.8733	49.27
1.0	-2056.1919	-2056.1147	48.43
1.5	-2056.4356	-2056.3605	47.14
2.0	-2056.6822	-2056.6111	44.63
2.5	-2056.9315	-2056.8650	41.72
3.0	-2057.1838	-2057.1236	37.80
3.5	-2057.4390	-2057.3832	34.99
4.0	-2057.6972	-2057.6463	31.91
4.5	-2057.9591	-2057.9125	29.24
5.0	-2058.2244	-2058.1821	26.50
5.5	-2058.4939	-2058.4559	23.81
5.9	-2058.7132	-2058.6787	21.64

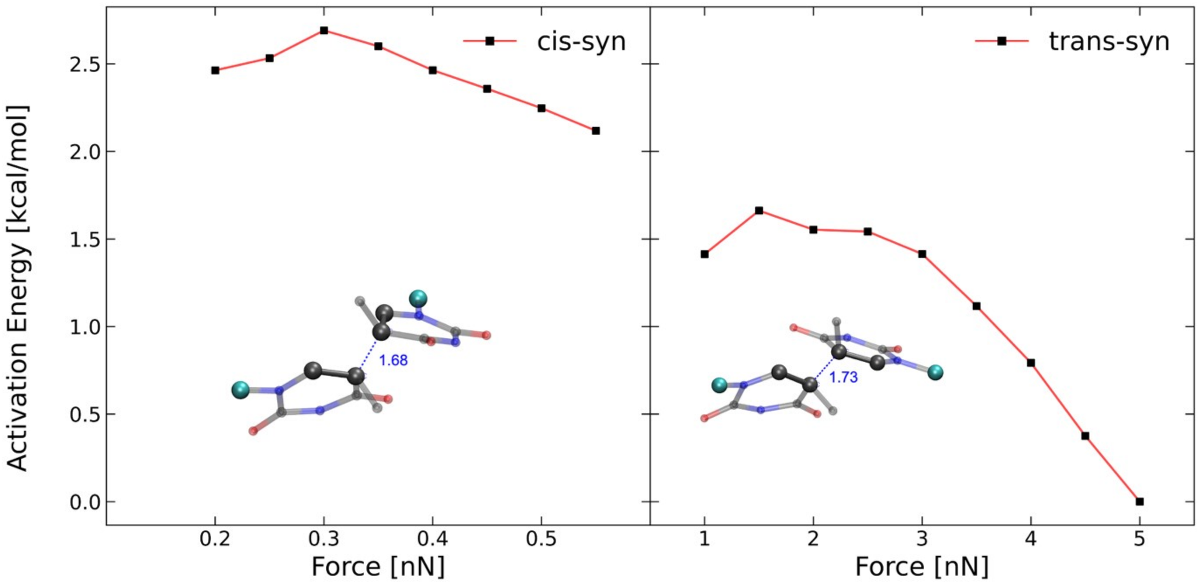

Figure S23. Barrier heights of the second bond breaking of the two *syn* CPD mechanophore isomers investigated with the FM-PES method. The displayed geometries show the transition state structures with the corresponding C-C atom distances computed at a force of 0.5 nN (*cis-syn*) and 2.0 nN (*trans-syn*), respectively.

Table S7. Calculated absolute potential energies V in atomic units for the reactant and transition state and the respective activation energy V_A in kcal/mol for the second reaction step of the ring opening reaction of *cis-syn* CPD.

	Reactant	Transition State	
Force [nN]	V[a.u.]	V[a.u.]	Potential activation energy V_A [kcal/mol]
0.20	-2055.7345	-2055.7306	2.46
0.25	-2055.7584	-2055.7543	2.53
0.30	-2055.7824	-2055.7781	2.69
0.35	-2055.8065	-2055.8024	2.60
0.40	-2055.8307	-2055.8268	2.46
0.45	-2055.8551	-2055.8514	2.36
0.50	-2055.9041	-2055.8760	2.25
0.55	-2055.8796	-2055.9008	2.12

Table S8. Calculated absolute potential energies V in atomic units for the reactant and transition state and the respective activation energy V_A in kcal/mol for the second reaction step of the ring opening reaction of *trans-syn* CPD.

	Reactant	Transition State	
Force [nN]	V[a.u.]	V[a.u.]	Potential activation energy V_A [kcal/mol]
1.0	-2056.1501	-2056.1479	1.41
1.5	-2056.4126	-2056.4099	1.66
2.0	-2056.6778	-2056.6754	1.55
2.5	-2056.9465	-2056.9441	1.54
3.0	-2057.2183	-2057.2161	1.41
3.5	-2057.4933	-2057.4915	1.12
4.0	-2057.7719	-2057.7706	0.79
4.5	-2058.0541	-2058.0536	0.38
5.0	-2058.3409	-2058.3409	0.00

References

1. H. E. Gottlieb, V. Kotlyar, A. Nudelman, *J. Org. Chem.*, 1997, **62**, 7512–7515.
2. T. Suzuki, H. Ota, Y. Namba, T. Fujino, *Chem. Pharm. Bull.*, 2019, **67**, 130–134.
3. G. R. Jones, Z. Li, A. Anastasaki, D. J. Lloyd, P. Wilson, Q. Zhang, D. M. Haddleton, *Macromolecules*, 2016, **49**, 483–489.
4. D. Yildiz, C. Baumann, A. Mikosch, A. J. C. Kuehne, A. Herrmann, R. Göstl, *Angew. Chem., Int. Ed.*, 2019, **58**, 12919–12923.
5. T. Suzuki, H. Ota, Y. Namba, T. Fujino, *Chem. Pharm. Bull.*, 2019, **67**, 130–134.
6. M. D. Hanwell, D. E. Curtis, D. C. Lonie, T. Vandermeersch, E. Zurek, G. R. Hutchison, *J. Cheminform.*, 2012, **4**, 1–17.
7. A. D. Becke, *J. Chem. Phys.*, 1992, **96**, 2155–2160.
8. C. Lee, W. Yang, R. G. Parr, *Phys. Rev. B*, 1988, **37**, 785–789.
9. S. H. Vosko, L. Wilk, M. Nusair, *Can. J. Phys.*, 1980, **58**, 1200–1211.
10. P. J. Stephens, F. J. Devlin, C. F. Chabalowski, M. J. Frisch, *J. Phys. Chem.*, 1994, **98**, 11623–11627.
11. R. Ditchfield, W. J. Hehre, J. A. Pople, *J. Chem. Phys.*, 1971, **54**, 724–728.
12. P. C. Hariharan, J. A. Pople, *Theor. Chim. Acta*, 1973, **28**, 213–222.
13. W. J. Hehre, R. Ditchfield, J. A. Pople, *J. Chem. Phys.*, 1972, **56**, 2257–2261.
14. M. K. Beyer, *J. Chem. Phys.*, 2000, **112**, 7307–7312.
15. M. K. Beyer, H. Clausen-Schaumann, *Chem. Rev.*, 2005, **105**, 2921–2948.
16. S. Seritan, C. Bannwarth, B. S. Fales, E. G. Hohenstein, S. I. L. Kokkila-Schumacher, N. Luehr, J. W. Snyder Jr., C. Song, A. V. Titov, I. S. Ufimtsev, T. J. Martínez, *J. Chem. Phys.*, 2020, **152**, 224110.
17. S. Seritan, C. Bannwarth, B. S. Fales, E. G. Hohenstein, C. M. Isborn, S. I. L. Kokkila-Schumacher, X. Li, F. Liu, N. Luehr, J. W. Snyder Jr., C. Song, A. V. Titov, I. S. Ufimtsev, L. Wang, T. J. Martínez, *WIREs Comput. Mol. Sci.*, 2021, **11**, e1494.
18. M. T. Ong, J. Leiding, H. Tao, A. M. Virshup, T. J. Martínez, *J. Am. Chem. Soc.*, 2009, **131**, 6377–6379.
19. J. Kästner, J. M. Carr, T. W. Keal, W. Thiel, A. Wander, P. Sherwood, *J. Phys. Chem. A*, 2009, **113**, 11856–11865.
20. S. Metz, J. Kästner, A. A. Sokol, T. W. Keal, P. Sherwood, *WIREs Comput. Mol. Sci.*, 2014, **4**, 101–110.
21. G. Henkelman, H. Jónsson, *J. Chem. Phys.*, 1999, **111**, 7010–7022.
22. J. Kästner, P. Sherwood, *J. Chem. Phys.*, 2008, **128**, 014106.
